# Endowing TADF luminophors with AIE properties through adjusting flexible dendrons for highly efficient solution-processed nondoped OLEDs†
†Electronic supplementary information (ESI) available: Synthesis and characterization details, NMR spectra of compounds, CV curves, SEM and AFM images. Photophysical properties and supplementary device performance. See DOI: 10.1039/d0sc02194f


**DOI:** 10.1039/d0sc02194f

**Published:** 2020-06-16

**Authors:** Dan Liu, Jing Yi Wei, Wen Wen Tian, Wei Jiang, Yue Ming Sun, Zheng Zhao, Ben Zhong Tang

**Affiliations:** a Jiangsu Province Hi-Tech Key Laboratory for Bio-Medical Research , Jiangsu Engineering Laboratory of Smart Carbon-Rich Materials and Device , School of Chemistry and Chemical Engineering , Southeast University , Nanjing , 211189 , China . Email: jiangw@seu.edu.cn; b Department of Chemistry , Hong Kong Branch of Chinese National Engineering Research, Center for Tissue Restoration and Reconstruction , The Hong Kong University of Science and Technology , Clear Water Bay , Kowloon , Hong Kong 999077 , China . Email: tangbenz@ust.hk

## Abstract

The amalgamation of thermally activated delayed fluorescence (TADF) and aggregation-induced emission (AIE) properties, termed AIE-TADF, is a promising strategy to design novel robust luminescent materials. Herein, we transform 2,3,4,5,6-penta(9*H*-carbazol-9-yl)benzonitrile (5CzBN) from an ACQ molecule into an AIEgen by simply decorating the 5CzBN core with alkyl chain-linked spirobifluorene dendrons. By increasing the number of flexible dendrons, these materials can not only show obvious AIE-TADF characteristics and uniform film morphology, but can also exhibit better resistance to isopropyl alcohol, which are beneficial to fully solution-processed OLEDs. Notably, 5CzBN-PSP shows great device efficiency with an external quantum efficiency (EQE), current efficiency and power efficiency of 20.1%, 58.7 cd A^–1^ and 46.2 lm W^–1^, respectively and achieved record-breaking efficiency in solution-processed nondoped OLEDs based on AIE emitters. This work demonstrates a general approach to explore new efficient emitters by the marriage of AIE and TADF which could potentially improve their performance in various areas.

## Abstract 2

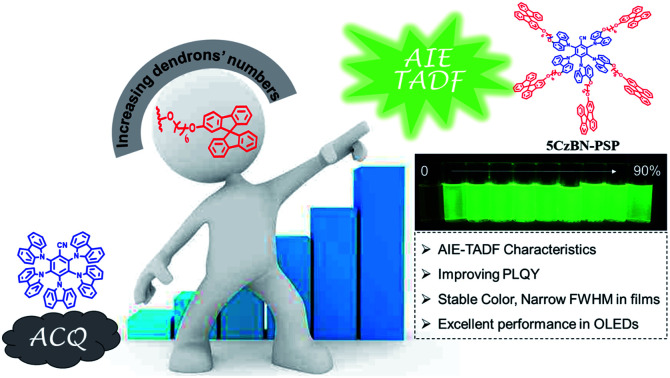
Construction of core–dendron TADF emitters systematically: changing the behaviour of chromophores from aggregation-caused quenching to aggregation induced emission to develop high-performance fully solution-processed nondoped OLEDs.

## Introduction

The reduction of the manufacturing cost of organic light-emitting diodes (OLEDs) remains one of the key challenges in the commercialization of OLED technology.^1^ Compared with vacuum deposition, solution-process techniques are more promising for large-area fabrication because of their high material utilization rate, simple manufacturing process, and easy operation.^2^–^4^ However, the efficiencies of solution-processed OLEDs are generally low, so there is an urgent need to develop more efficient luminescent materials.^5^,^6^ After the pioneering work by Adachi and co-workers, thermally activated delayed fluorescent (TADF) materials have been explored as the most promising third generation emitters followed by conventional fluorescent materials, and phosphorescent heavy-metal complexes.^7^–^9^ In addition, several small-molecule TADF emitters have been employed in solution-processed OLEDs and achieved relatively high device performance.^10^–^12^ It is vital to emphasize that the majority of TADF emitters are doped in appropriate host matrices to weaken the intermolecular interactions and exciton quenching.^13^,^14^ As is known, strong luminescence of conventional organic fluorophores in dilute solution is normally weakened or quenched in their aggregated states, and there is no exception for most TADF materials.^15^–^17^ Although long-lived triplet excitons in TADF molecules can be up-converted into radiable singlet excitons through the reverse intersystem crossing (RISC) process, the lower RISC rate (*k*_RISC_) inevitably results in quenching of many triplet excitons in the aggregated state by triplet–triplet annihilation (TTA), singlet–triplet annihilation (STA) and triplet-polaron annihilation (TPA).^18^,^19^ The aggregation-caused quenching (ACQ) effect seriously limits their application and reduces the device performance. Fortunately, Tang and co-workers have reported a series of new luminogens with aggregation-induced emission (AIE) properties since 2001.^20^–^22^ These AIE compounds effectively overcome the drawbacks of ACQ and achieve efficient solid-state luminescence. The enhanced photoluminescence quantum yield (PLQY) in the aggregation state enables the performances of nondoped OLEDs to be improved.^23^–^25^

Based on the above research, TADF materials can harvest both singlet and triplet excitons to achieve theoretical 100% internal quantum efficiency, and AIE luminogens are favored to attain efficient emission in their condensed solid states. Therefore, integrating TADF emitters with the AIE nature could be a feasible strategy to develop efficient solution-processed nondoped OLEDs.^26^–^28^ With this design concept, various types of AIE-TADF materials have been reported in recent years by Tang and Chi, *et al.*^29^–^31^ Despite the nondoped devices exhibiting improved electroluminescence efficiencies, further utilization of these emitters in the solution process has been rarely explored.^32^ This is mainly due to many inevitable challenges that need to be overcome. (i) Most of the AIE-TADF materials have poor solubility, making them unsuitable for nondoped solution processes. (ii) High crystallization of AIE-TADF materials in film states can lead to the generation of dark current and exciton traps, eventually reducing device performance. (iii) In a long run, the common AIE-TADF materials cannot resist the erosion of solvent used for the processing of upper layers, which limited their further development in fully solution-processed OLEDs. Therefore, there is room for further development to extend the structural diversity of solution-processable AIE-TADF materials and enhance their device performances.

**Fig. 1:**
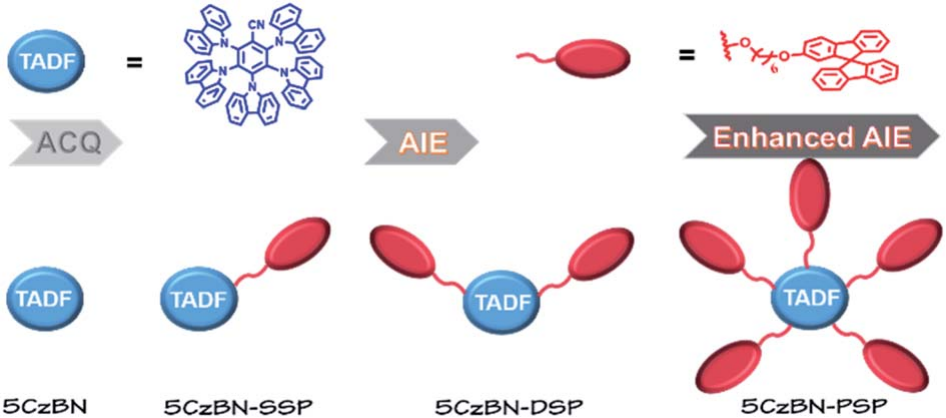
Concept of the core–dendron system in nondoped emissive layers and chemical structures of 5CzBN, 5CzBN-SSP, 5CzBN-DSP and 5CzBN-PSP.

In this contribution, a novel type of AIE-TADF molecule was developed by constructing a core–dendron structure, which has not been reported in previous research. We designed and synthesized three AIE-TADF molecules named 5CzBN-SSP, 5CzBN-DSP and 5CzBN-PSP (Fig. 1). The core molecule was 2,3,4,5,6-penta(9*H*-carbazol-9-yl)benzonitrile (5CzBN), which exhibits TADF characteristics.^33^ The flexible dendrons were different numbers of alkyl chain-linked spirobifluorene. Owing to increased molecular weight and solubilizing alkyl chains, these materials showed a good film morphology and are suitable for solution processes. More interestingly, although the core 5CzBN and the dendron spirobifluorene displayed ACQ properties, core–dendron materials demonstrated the AIE phenomenon. With increasing the number of flexible branches, the compounds showed better solubility, a more smooth surface morphology, more obvious AIE features and higher photoluminescence quantum yields (PLQYs). By employing these AIE-TADF materials (5CzBN-SSP, 5CzBN-DSP, and 5CzBN-PSP) as emitters, fully solution-processed nondoped OLEDs achieved high external quantum efficiencies (EQE) of 7.3%, 13.9% and 20.1%, which far exceed those of the fully solution processed OLEDs based on 5CzBN. Furthermore, it is worth mentioning that the OLED based on 5CzBN-PSP showed a record-breaking external EQE of 20.1% in the area of solution-processed nondoped OLEDs based on AIE emitters so far. The core–dendron system will provide a good candidate for highly efficient solution-processed nondoped OLEDs.

## Results and discussion

**Scheme 1:**
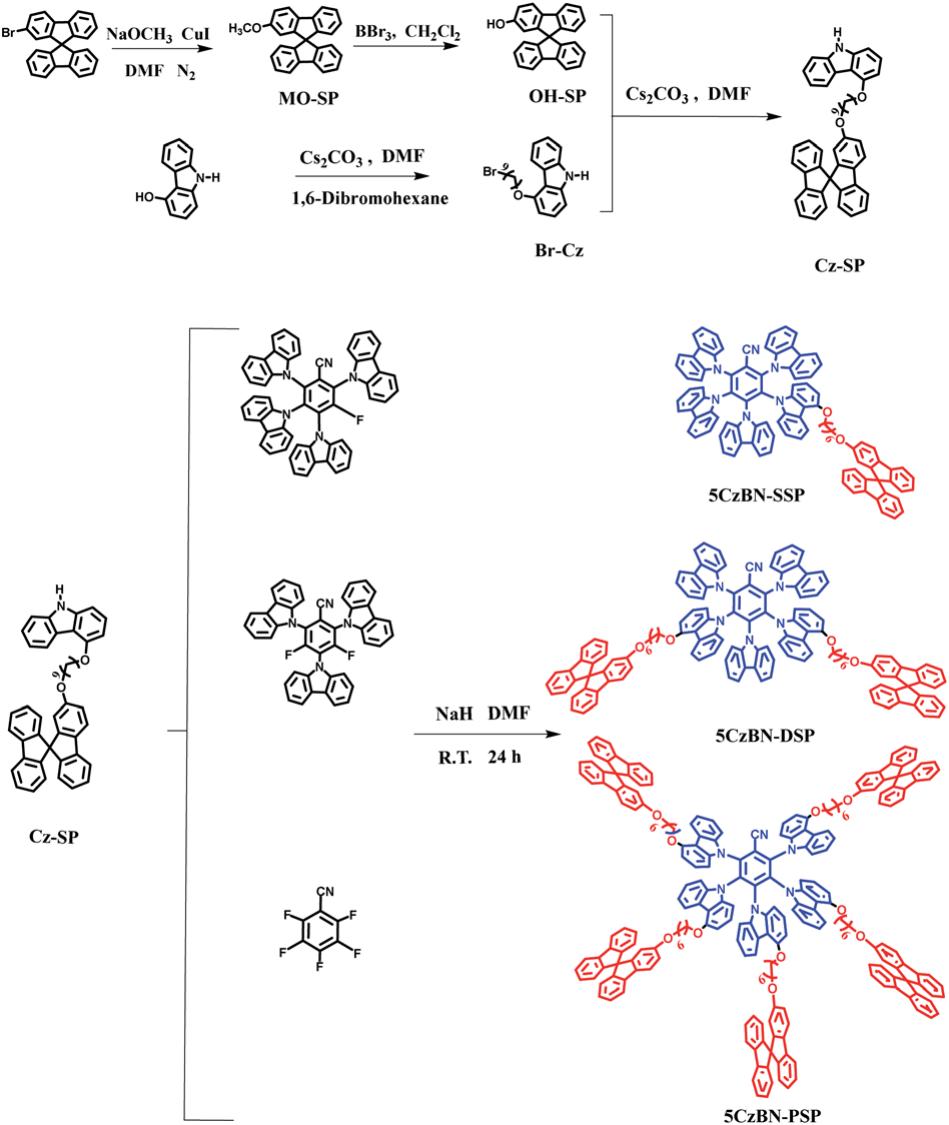
Synthetic routes and chemical structures of 5CzBN-SSP, 5CzBN-DSP and 5CzBN-PSP.

2,3,4,5,6-Penta(9*H*-carbazol-9-yl)benzonitrile (5CzBN) was selected as the TADF emitter unit, and alkyl chain-linked spirobifluorene was used as the functional dendron. The preparation of the target compounds 3-(4-((6-(9,9′-spirobi[fluoren]-2-yloxy)hexyl)oxy)-9*H*-carbazol-9-yl)-2,4,5,6-tetra(9*H*-carbazol-9-yl)benzonitrile (5CzBN-SSP), 3,5-bis(4-((6-(9,9′-spirobi[fluoren]-2-yloxy)hexyl)oxy)-9*H*-carbazol-9-yl)-2,4,6-tri(9*H*-carbazol-9-yl)benzonitrile (5CzBN-DSP) and 2,3,4,5,6-pentakis(4-((6-(9,9′-spirobi[fluoren]-2-yloxy)hexyl)oxy)-9H-carbazol-9-yl)benzonitrile (5CzBN-PSP) was carried out by following the synthetic procedure shown in Schemes 1 and S1.† All the AIE-TADF compounds are synthesized through catalyst free aromatic nucleophilic substitution reactions. Before device fabrication, the target product was separated and purified using a silica gel column.

The thermal stability properties of 5CzBN-SSP, 5CzBN-DSP and 5CzBN-PSP were characterized by thermal gravimetric analysis (TGA) and differential scanning calorimetry (DSC) under a nitrogen atmosphere at a heating rate of 10 °C min^–1^. As is shown in Fig. S1,† the decomposition temperatures (*T*_d_) with 5% weight loss of 5CzBN-SSP, 5CzBN-DSP and 5CzBN-PSP were 406.2 °C, 394.7 °C and 393.8 °C, which demonstrated that all three emitters have high thermal stability. The glass transition temperatures (*T*_g_) of 5CzBN-SSP, 5CzBN-DSP and 5CzBN-PSP were 166.8 °C, 163.7 °C and 144.2 °C, respectively. And no crystal domains were formed during the thermal annealing process. The high *T*_g_ values ensured that the three materials can form uniform amorphous films during the solution process even though the values of *T*_d_ and *T*_g_ decrease to some extent when the number of branches gradually increases. Besides, we also investigated the morphology of the nondoped films by AFM and SEM measurements (Fig. S2 and S3†). The films of 5CzBN-SSP, 5CzBN-DSP and 5CzBN-PSP are very smooth and the root-mean-square values are 0.50, 0.43 and 0.38 nm, respectively. The values are better than that of 5CzBN (0.74 nm).^34^ In addition, the SEM images of the emission layers were collected from various parts of the films (at various magnifications of 1000 and 4000). In Fig. S3,† an obvious trend can be observed which showed that the spin-coated emissive layer exhibited a more homogeneous film with fewer defects as the number of dendrons increased. However, the solution-processed 5CzBN films showed pinholes and partially crystalline granulates over the entire film surface. This is also consistent with the AFM measurements with gradually reduced roughness. Furthermore, the SEM images of the powder were also obtained to further support the above views; 5CzBN-SSP, 5CzBN-DSP and 5CzBN-PSP showed gradually uniformly distributed spherical particles, while 5CzBN exhibited aggregated bulk particles tending to have a crystalline state. The XRD plots also confirm the amorphous nature of the dendron derivatives in the solid state (Fig. S4†). This indicated that all three materials can form uniform amorphous films through the solution process. And it is also convincing that the flexible chain-linked spirobifluorene units can remarkably decrease the crystallization tendency and suppress the aggregation between the molecules. The better solubility and morphology are more beneficial for the fabrication of highly efficient fully solution processed OLEDs.

**Fig. 2:**
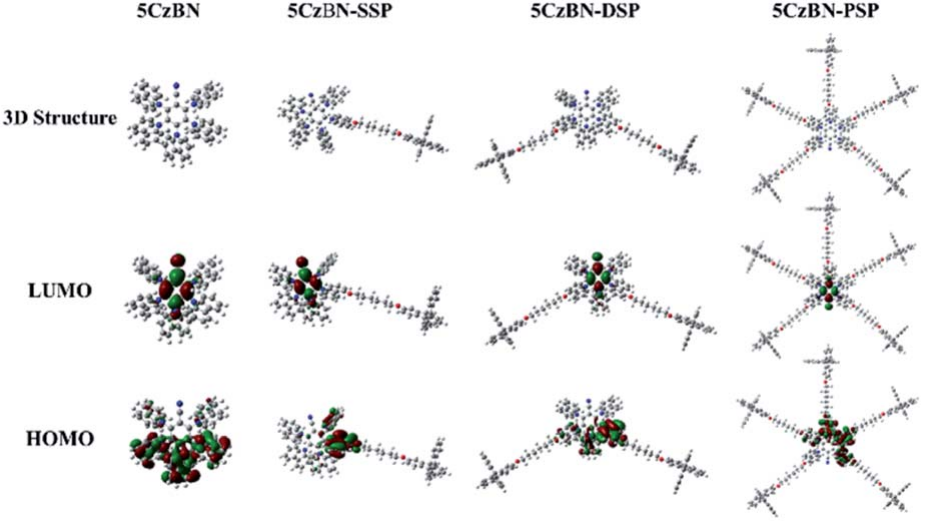
Optimized geometries and electron cloud distribution maps of 5CzBN, 5CzBN-SSP, 5CzBN-DSP and 5CzBN-PSP.

The electronic properties of 5CzBN, 5CzBN-SSP, 5CzBN-DSP and 5CzBN-PSP were researched using density functional theory (DFT) calculations at the B3LYP theory level with the 6-31G(d) as the basis set. The calculated results are shown in Fig. 2; the highest occupied molecular orbital (HOMO) electron clouds of all four materials are mainly located on the carbazolyl group of the emissive core, while the lowest unoccupied molecular orbital (LUMO) electron clouds are mainly located on the electron-deficient benzonitrile units. Compared with the 5CzBN emitter (–5.54 eV), the HOMO levels and the calculated energy gap between S_1_ and T_1_ (Δ*E*_ST_) of 5CzBN-SSP, 5CzBN-DSP and 5CzBN-PSP are –5.36 eV/0.15 eV, –5.35 eV/0.13 eV and –5.25 eV/0.11 eV, respectively. They changed regularly with the increasing of the number of electron-donating spirobifluorene groups. Thus, it is concluded that more spirobifluorene dendrons can make the levels of HOMO shallower. In addition, the smaller Δ*E*_ST_ ensures efficient RISC and subsequently efficient TADF emission. The electrochemical properties were measured by using cyclic voltammetry (CV). As is shown in Fig. S5,† the energy levels of the HOMO of 5CzBN-SSP, 5CzBN-DSP and 5CzBN-PSP are –5.25, –5.23 and –5.22 eV, respectively. And the results matched well with those of the theoretical simulations. According to the equation *E*_LUMO_ = *E*_HOMO_ + *E*_g_, where *E*_g_ is the optical band gap calculated from absorption spectra, the energy of the LUMO of 5CzBN-SSP, 5CzBN-DSP and 5CzBN-PSP is –2.52, –2.48 and –2.52 eV, respectively (Table 1). Therefore, the shallower HOMO levels of these 5CzBN derivatives are close to the energy level of the hole transport layer PEDOT:PSS (–5.2 eV), which would facilitate the hole injection into the emitting layer. Furthermore, we measured the redox curves after 100 cycles (Fig. S5†), and the peak potential and peak current remain unchanged. So these compounds show good electrochemical stability,^35^–^37^ which is beneficial to long-term device operation.

**Table 1:** Basic thermal, photophysical and electrochemical parameters of 5CzBN-SSP, 5CzBN-DSP and 5CzBN-PSP

Compounds	*T*_g_/*T*_d_^*a*^ [°C]	*λ*_abs_ ^*b*^ [nm]	*λ*_em_ ^*c*^ ^/^ ^*d*^ ^/^ ^*e*^ [nm]	*τ*_p_ ^*f*^/*τ*_d_^*f*^	FWHM^*c*^ ^/^^*d*^ ^/^^*e*^ [nm]	*E*_g_ ^*g*^ (eV)	T_1_^*h*^ [eV]	HOMO^*j*^ [eV]	LUMO^*k*^ [eV]	S_1_^*i*^ [eV]	Δ*E*_ST_^*l*^ ^/^^*m*^ [eV]
5CzBN-SSP	166.8/406.2	229/275/310	515/502/473	38 ns/3.1 μs	96/81/85	2.73	2.80	–5.25	–2.52	2.96	0.16/0.15
5CzBN-DSP	163.7/394.7	230/285/310	517/504/476	56 ns/3.8 μs	88/78/80	2.75	2.78	–5.23	–2.48	2.93	0.15/0.13
5CzBN-PSP	144.2/393.8	230/288/312	523/504/480	77 ns/4.5 μs	110/71/74	2.70	2.74	–5.22	–2.52	2.87	0.13/0.11

^*a*^ *T*_d_ corresponds to 5% weight loss.

^*b*^Measured in CH_2_Cl_2_ solution at room temperature.

^*c*^Measured in CH_2_Cl_2_ solution at 298 K.

^*d*^Measured in the neat film at 298 K.

^*e*^Measured in toluene solution at 298 K.

^*f*^Prompt and delayed fluorescence lifetimes of the neat film at room temperature.

^*g*^Estimated from the absorption edges in the neat film.

^*h*^Estimated from the onset of the phosphorescence spectra in toluene at 77 K.

^*i*^Calculated from the onset of the fluorescence spectra in toluene at 298 K.

^*j*^The HOMO energies were determined from the onset potentials of oxidation with regard to the energy level of ferrocene (4.8 eV below vacuum).

^*k*^Calculated from the HOMO and optical energy gap.

^*l*^Difference between the S_1_ energy and T_1_ energy.

^*m*^Results of the DFT calculations.

**Fig. 3:**
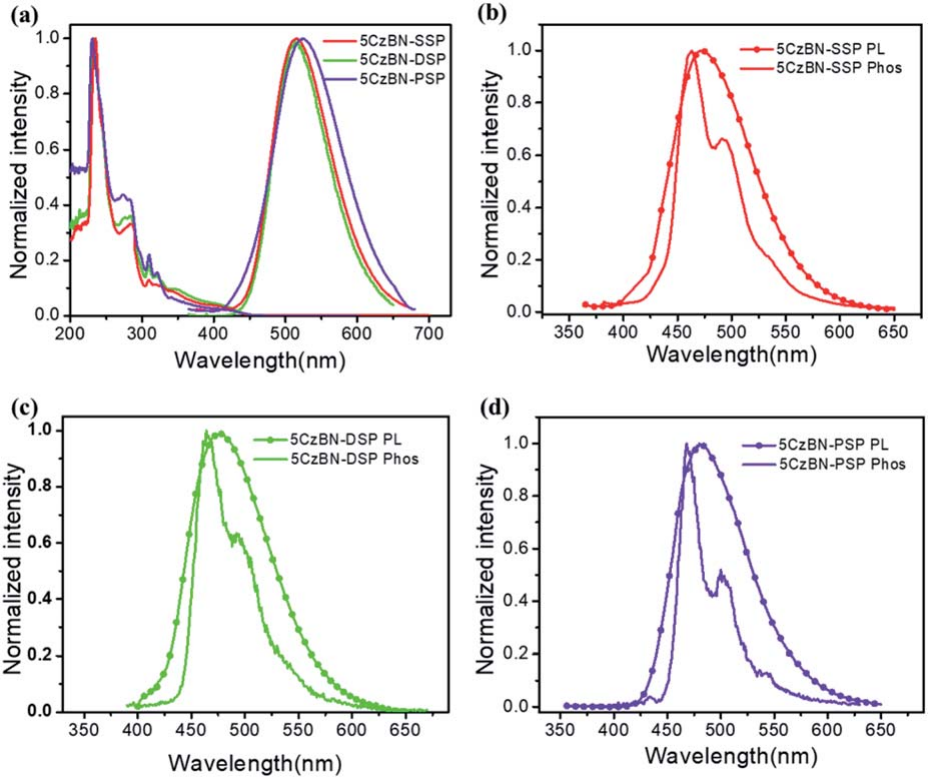
(a) UV-vis absorption and fluorescence spectra of 5CzBN-SSP, 5CzBN-DSP and 5CzBN-PSP in CH_2_Cl_2_, (b) fluorescence and phosphorescence spectra of 5CzBN-SSP in toluene, (c) fluorescence and phosphorescence spectra of 5CzBN-DSP in toluene, and (d) fluorescence and phosphorescence spectra of 5CzBN-PSP in toluene.

The photophysical properties of the three TADF materials were measured using an ultraviolet-visible (UV-Vis) absorption and fluorescence spectrometer. As is depicted in Fig. 3 and S6,† the absorption bands at 300–400 nm can be attributed to the π–π* band of Cz units, and a band at about 400–450 nm resulted from the ICT transitions.^38^ The energy band gaps (*E*_g_) of the three materials were calculated to be 2.73, 2.75, and 2.77 eV from the the absorption edge. Also, the photoluminescence (PL) and phosphorescence (Phos) spectra were measured in toluene at room temperature and at 77 K. All data are summarized in Table 1. The PL spectra of 5CzBN-SSP, 5CzBN-DSP and 5CzBN-PSP in toluene showed emission bands at 473, 476 and 480 nm, respectively while in CH_2_Cl_2_ they showed at 515, 517 and 523 nm due to increased solvent polarity. The broad and featureless peaks revealed that the S_1_ states of the three compounds are charge-transfer (CT) states. The S_1_ energy levels of 5CzBN-SSP, 5CzBN-DSP and 5CzBN-PSP were calculated to be 2.96, 2.93 and 2.87 eV in toluene. The low-temperature PL at 77 K was monitored to evaluate the triplet energy levels (T_1_) of 5CzBN-SSP, 5CzBN-DSP and 5CzBN-PSP, yielding values of 2.80, 2.78 and 2.74 eV, respectively. Subsequently, Δ*E*_ST_ values of 0.16, 0.15 and 0.13 eV were respectively obtained for 5CzBN-SSP, 5CzBN-DSP and 5CzBN-PSP by following the literature methods.^39^ The PL spectra and UV-vis spectra of the spin-coated films are shown in Fig. S6;† the spin-coated films exhibited emission peaks at 502 nm, 504 nm and 504 nm. This demonstrated that alkyl-linked spirobifluorene (SP) can effectively reduce the intermolecular interaction of emissive cores and then further maintain the colour of the 5CzBN core. In addition, the FWHM values of 5CzBN-SSP, 5CzBN-DSP and 5CzBN-PSP are 81 nm, 78 nm and 71 nm, respectively. The narrower FWHM of 5CzBN-PSP is attributed to sufficient encapsulation by the peripheral alkyl-linked spirobifluorenes. Furthermore, all three materials exhibit bathochromic shifts accompanied by the increase of solvent polarity (Fig. S7†). The large solvatochromic shift indicates that the three compounds exhibit typical ICT characteristics.^40^ Notably, the wavelength of 5CzBN-PSP is shifted by a smaller amount than those of 5CzBN-SSP and 5CzBN-DSP due to sufficient encapsulation by large numbers of spirobifluorene dendrons. More SP dendrons which acted as the steric shield, weakened the response to environmental polarity.

**Fig. 4:**
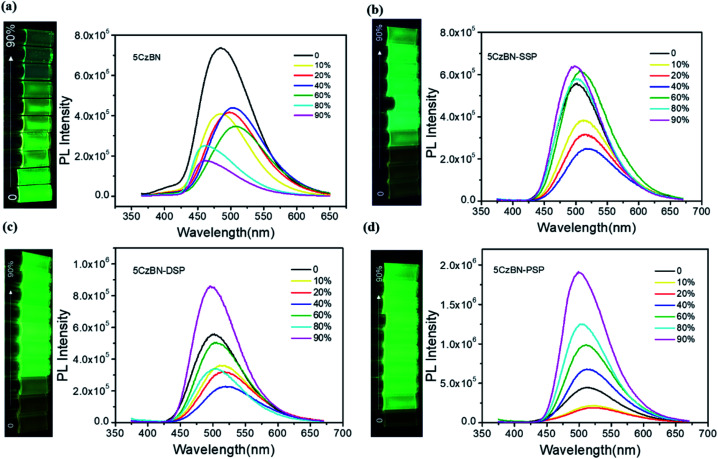
PL spectra and emission images of (a) 5CzBN, (b) 5CzBN-SSP, (c) 5CzBN-DSP, and (d) 5CzBN-PSP in THF–water mixtures (10^–5^ M) with different fractions of water, respectively, under 365 nm UV irradiation.

**Fig. 5:**
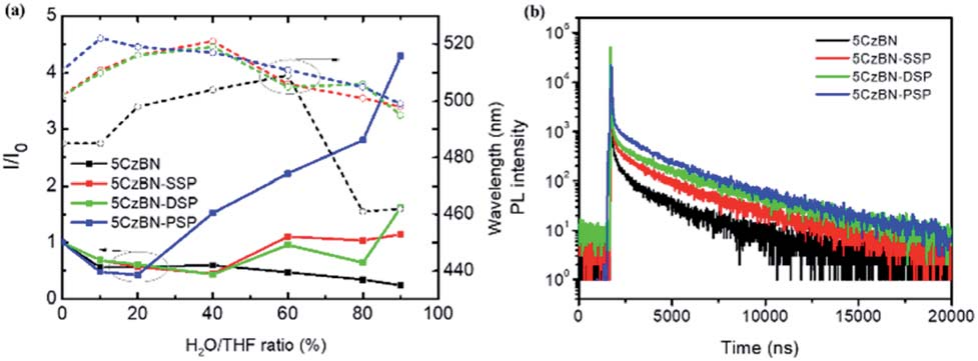
(a) The plots of the fluorescence ratio (*I*/*I*_0_) and peak wavelength *versus* the water volume fraction. (b) Transient PL decay spectra in the neat film measured at room temperature for 5CzBN-SSP, 5CzBN-DSP and 5CzBN-PSP.

In order to explore the AIE features of these materials, the PL intensity in the mixture of tetrahydrofuran (THF) and deionized water with various deionized water ratio fractions was measured and is depicted in Fig. 4 and 5a. For 5CzBN, the PL intensity showed an overall slow decline when water fractions (*f*_w_) increased from 0% to 90%, even if there is a slight rise between 20–40% and 60–70%. This ACQ phenomenon can be attributed to the increased ISC rate and twisted intramolecular charge transfer (TICT) process, which makes the exciton quenching non-radiative. In pure THF solution, these molecules show weak fluorescence. And when the water fractions *f*_w_ were increased, with a rise in polarity the preferential solvation of the TICT state decreases the energy gap between the TICT state and the triplet. In addition, the rate of ISC from the TICT singlet state increases as the singlet–triplet energy gap decreases, and therefore the dark TICT state exhibited weaker emission, that is, PL quenching. However, by increasing the number of flexible dendrons, the PL intensity of the three compounds showed different trends (AIE trends), especially for 5CzBN-PSP. 5CzBN-PSP achieved a significant fluorescence enhancement from 20% to 90%, and the PL intensity in 90% mixtures is more than 4 times stronger than the initial intensity (*I*_0_). 5CzBN-SSP and 5CzBN-DSP also exhibited a slight enhancement in the PL intensity when *f*_w_ was increased from 40% to 90%. Thus, these results provide significant evidence to prove the AIE properties of the three compounds, though the degree of AIE enhancement differed for each molecule.^41^–^43^ More interestingly, the peak of 5CzBN underwent a red-shift with increasing addition of water in the mixed solvent until molecular aggregation was induced to form nano-aggregates. Thereafter, the emission demonstrated a blue-shift until the concentration was increased to 90%. The same phenomenon can be observed in 5CzBN-SSP, 5CzBN-DSP and 5CzBN-PSP. And this is also caused by a TICT process.^44^ Before the generation of aggregates, solvatochromism accounts for the red-shift of the emission peak with increasing solvent polarity. In contrast, after the generation of nano-aggregates, the intramolecular rotation is restricted and the local environment becomes less polar, thereby resulting in a blueshift in the emission color. The curves of the emission wavelength *versus* the water fraction are shown obviously in Fig. 5a. Some differences can also be found in this picture. With the increase in the number of spirobifluorene branches, the change in the emission wavelength becomes smaller and smaller. This suggested that with increasing the number of dendrons, the TICT process is suppressed efficiently, and the influence of the environmental polarity on the emission color of the materials becomes weak. Simultaneously, the AIE feature of dendrimers is more outstanding. From these results, it was confirmed that the AIE phenomenon that does not exist in 5CzBN is emerging and enhanced in 5CzBN-SSP, 5CzBN-DSP and 5CzBN-PSP. Furthermore, it can also be anticipated that the great AIE character for 5CzBN-PSP should be more favourable for its use as an emitter for highly efficient solution-processed nondoped OLEDs.^45^

To better understand the TADF features of the three compounds, the transient PL decay profiles were measured for the films. As is depicted in Fig. 5, the PL decays of the three molecules exhibit bi-exponential decays which show prompt and delayed lifetimes of the components, fully demonstrating that 5CzBN, 5CzBN-SSP, 5CzBN-DSP and 5CzBN-PSP are apparent TADF materials.^41^ And the data of the three compounds are presented in Table 1. The transient lifetime of the three compounds is due to the conventional fluorescence from S_1_ to S_0_. The delayed lifetime is attributed to the reverse intersystem crossing (RISC) process from the non-radiative T_1_ state to the radiative S_1_ state and finally to S_0_ because of the small Δ*E*_ST_. Thus, the PL decay revealed that the three molecules have TADF features. The delayed fluorescence lifetimes became longer with increasing the number of flexible dendrons. Besides, the PLQYs of 5CzBN-SSP, 5CzBN-DSP and 5CzBN-PSP in the neat films were measured to analyse the emission features. As shown in Table 2, the PLQYs of 5CzBN-SSP, 5CzBN-DSP and 5CzBN-PSP are 38.0%, 45.7%, and 69.6%, respectively. 5CzBN-PSP has a higher PLQY than the other materials. This is also consistent with the better AIE phenomenon of 5CzBN-PSP. This depicted that increasing the number of alkyl chain-linked dendrons can improve the PLQY by suppressing the core's collision and is more beneficial for the fabrication of excellent nondoped fully solution-processed OLEDs.

**Table 2:** Summary of solution-processed OLEDs

EML	*V*_on_ ^*a*^ [V]	*L*_max_ ^*b*^ [cd m^–2^]	*Φ*_F_ ^*c*^ [%]	CE_max_^*d*^ [cd A^–1^]	PE_max_^*e*^ [lm W^–1^]	EQE_max_^*f*^ [%]	CIE(*x*, *y*)^*g*^
5CzBN-SSP	3.4	5400	38.0	21.9	17.2	7.3	[0.28, 0.54]
5CzBN-DSP	3.2	14 800	45.7	40.7	31.9	13.9	[0.27, 0.54]
5CzBN-PSP	3.1	13 700	69.6	58.7	46.2	20.1	[0.27, 0.53]

^*a*^ *V*_on_ = turn-on voltage at 1 cd m^–2^.

^*b*^ *L*_max_ = maximum luminance.

^*c*^ *Φ*_F_ = the photoluminescence quantum yield of neat films.

^*d*^CE_max_ = maximum current efficiency.

^*e*^PE_max_ = maximum power efficiency.

^*f*^EQE_max_ = maximum external quantum efficiency.

^*g*^CIE = the Commission Internationale de L'Eclairage coordinates at 10 V.

**Fig. 6:**
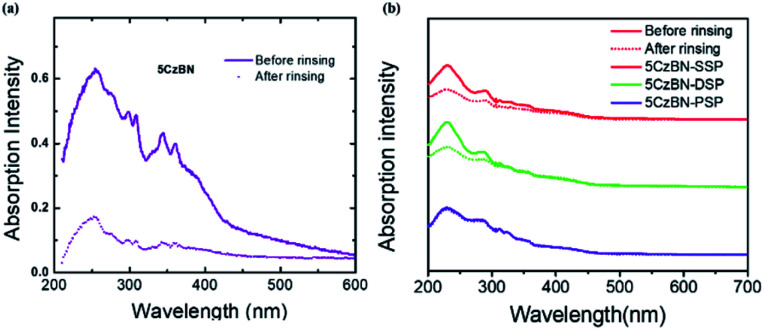
Absorption spectra before and after rinsing with isopropanol of 5CzBN (a), 5CzBN-SSP, 5CzBN-DSP and 5CzBN-PSP (b).

Before fabricating fully solution processed OLEDs, alcohol resistance was measured by UV-Vis absorption spectroscopy.^46^Fig. 6 shows the variations in the absorption intensity of 5CzBN, 5CzBN-SSP, 5CzBN-DSP and 5CzBN-PSP before and after spin-rinsing with isopropanol alcohol; isopropanol alcohol would be used to process the adjacent electron-transport layer (ETL). The absorption intensity of 5CzBN-SSP, 5CzBN-DSP and 5CzBN-PSP remained nearly constant while the 5CzBN film is rinsed thoroughly with isopropanol alcohol. So this proves that the core would be encapsulated more sufficiently by introducing more alkyl chain-linked spirobifluorene dendrons. The compounds are more resistant to isopropanol alcohol. In other words, by increasing the number of dendrons, the materials can adequately prevent the redissolution by isopropanol.

In order to investigate the electro-luminescence (EL) properties of the three AIE-TADF materials, nondoped OLED devices have been fabricated by using the solution-processed nondoped emission layer (EML) and electron-transport layer (POT2T: ((1,3,5-triazine-2,4,6-triyl)tris(benzene-3,1-diyl))tris(diphenylphosphineoxide)). The structures of the three devices were indium-tin oxide (ITO)/PEDOT:PSS (40 nm, spin-coated)/EML (40 nm, spin-coated)/POT2T (40 nm, spin-coated)/Cs_2_CO_3_ (2 nm)/Al (100 nm). In this structure, PEDOT:PSS and POT2T served as the hole and electron transport materials, and Cs_2_CO_3_ was used as an electron injection layer. The schematic energy-level diagrams and the fabrication process of the devices are shown in Fig. S8.† These materials are all spin-coated to serve as the emission layer.

**Fig. 7:**
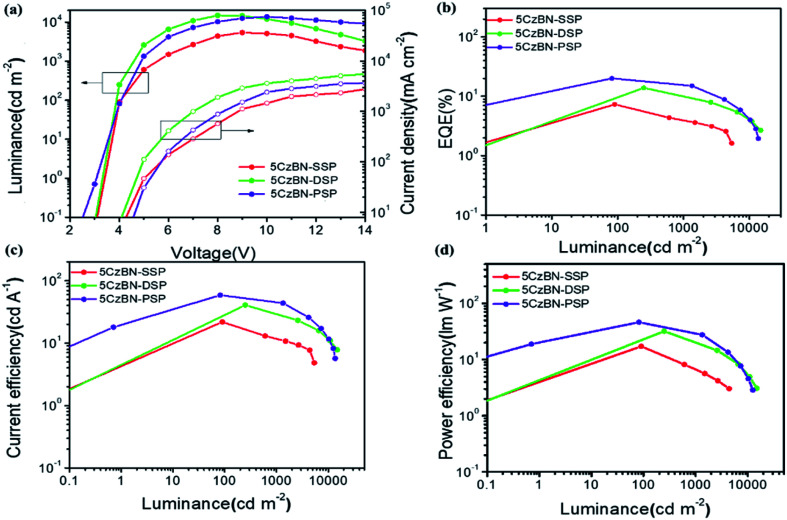
(a) The plots of the fluorescence ratio (*I*/*I*_0_) and peak wavelength *versus* the water volume fraction. (b) Transient PL decay spectra in the neat film measured at room temperature for 5CzBN-SSP, 5CzBN-DSP and 5CzBN-PSP.

**Fig. 8:**
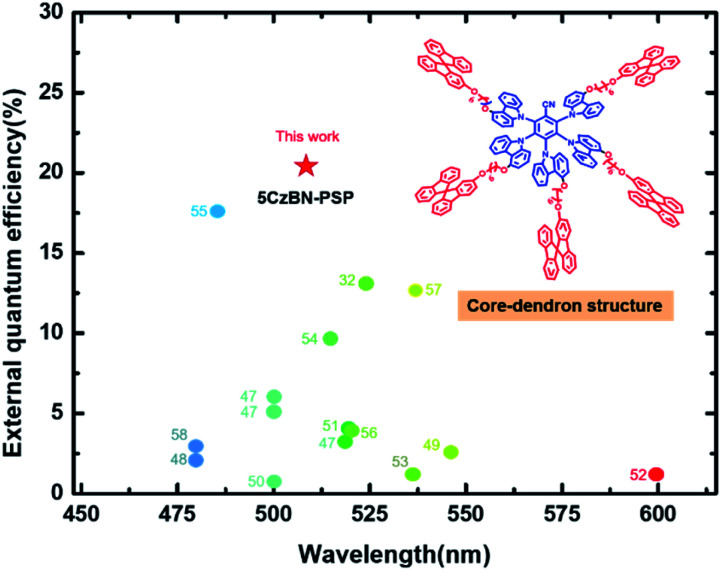
The summarized maximum external quantum efficiencies (EQE_max_) *versus* the electroluminescence wavelength in solution-processed non-doped AIE OLEDs.^32^,^47^–^58^ The numbers correspond to references from which values were obtained.

The devices with the three compounds showed green emission with a peak positioned at 508 nm. Fig. 7 shows the luminance–voltage–current density (*L*–*V*–*J*) and other EL properties of these devices, and the data of the devices are summarized in Table 2. Fig. S9† shows that the EL spectra of 5CzBN-SSP, 5CzBN-DSP and 5CzBN-PSP have similar emission. The FWHM values are 90 nm, 88 nm and 84 nm, respectively. 5CzBN-PSP exhibited a narrower FWHM because of weaker interactions between 5CzBN cores. The turn-on voltages of 5CzBN-SSP, 5CzBN-DSP and 5CzBN-PSP are 3.4 V, 3.2 V and 3.1 V. The maximum luminance, CE, PE, and EQE of 5CzBN-PSP are 13 700 cd m^–2^,58.7 cd A^–1^, 46.2 lm W^–1^ and 20.1%, respectively (Table 2), which were the highest efficiency in the reported solution-processed AIE OLEDs.^47^–^58^ All reported solution process nondoped AIE OLED performances are summarized in Fig. 8 and Table S1;† it is worth mentioning that there are very few reports on fully solution-processed AIE OLEDs and our work breaks the record of efficiencies of solution-processed nondoped AIE OLEDs. It indicated that AIE-TADF dendrimers can suppress exciton quenching of the emitter core,^59^ and good film-forming properties and high efficiencies can be achieved by increasing the number of branches to the core of the materials. All in all, regulating peripheral dendritic branched chains provided a new method for design of novel AIE-TADF materials. And inspired by the excellent device performance, this work would provide critical guidelines for design of solution-processable materials used in the fully wet-processed optoelectronic filed.

## Conclusions

In summary, a series of new core–dendron materials with AIE-TADF characteristics have been synthesized and studied by adjusting the number of alkyl chain-linked spirobifluorene dendrons. With the increasing number of flexible dendrons, 5CzBN-PSP exhibited both significant TADF and AIE features. Furthermore, it showed better resistance to isopropyl alcohol than 5CzBN-SSP and 5CzBN-DSP; the device with 5CzBN-PSP achieved a maximum external quantum efficiency of 20.1%, and current and power efficiencies of 58.7 cd A^–1^ and 46.2 lm W^–1^ and showed more efficient performance than fully solution-processed OLEDs based on traditional TADF materials. Our research indicated that adjusting the number of the alkyl-chain linked spirobifluorene dendrons attached to the TADF cores can transform common fluorophores even ACQ molecules into new AIE molecules. This work thus opens up a new route to design new AIE-TADF emitters with efficient performance in optoelectronic applications.

## Experimental section

### General methods

All solvents and materials were used as received from commercial sources without further purification. Anhydrification of THF solvent was carried out according to standard procedures. ^1^H NMR and ^13^C NMR spectra were recorded on a BRUKER AMX 600 MHz instrument. Elemental analysis was carried out using an Elementar Vario EL CHN elemental analyzer. Mass spectrometry was performed with a Thermo Electron Corporation Finnigan LTQ mass spectrometer. The UV-Vis absorption spectra of the compounds were measured using a SHIMADZU UV-2450. The absolute PLQYs of these materials were measured with a Hamamatsu Quantaurus-QY C11347 spectrometer. The photoluminescence emission spectra were recorded on a HORIBA FLUOROMAX-4 and liquid nitrogen was placed into an optical Dewar flask for low temperature (77 K) photophysical measurements. Thermogravimetric analysis (TGA) and differential scanning calorimetry (DSC) curves were recorded with a Netzsch simultaneous thermal analyzer (STA) system (STA409PC) and DSC 2910 modulated calorimeter under a dry nitrogen gas flow at a heating rate of 10 °C min^–1^. Cyclic voltammetry (CV) was performed on a CHI750C voltammetric analyzer in a typical three-electrode cell with a platinum plate working electrode, a platinum wire counter electrode and a silver wire reference electrode. The supporting electrolyte was tetrabutylammonium hexafluorophosphate (0.1 M) and ferrocene was selected as the internal standard. AFM (Seiko Instruments, SPA-400) was used to measure the film surface morphology. The measured pure film were formed by spin-coating and the solvent is 1,2-dichloroethane. The optimized structure was calculated using Gaussian 09 at the B3LYP functional with 6-31G(d) basis sets. The molecular orbitals were visualized using Gaussview 5.0.

### Device fabrication and measurements

ITO-coated glass substrates were rinsed with deionized water and then ultrasonicated sequentially in acetone and ethanol. Before device fabrication, the ITO substrate was treated in a UV-ozone oven for 20 min. Then a 40 nm thick poly(3,4-ethylenedioxythiophene):poly(styrenesulfonate) (PEDOT:PSS) film was first deposited on the pre-cleaned ITO glass substrates and baked at 150 °C for 10 min. Then, an EML with a thickness of about 40 nm was spin-coated from 1,2-dichloroethane solution onto the PEDOT:PSS layer and annealed at 100 °C for 30 min to remove the residual solvent in a N_2_ atmosphere. PO-T2T was spin-coated from isopropanol solution as the electron transporting layer, respectively. Finally, 2 nm thick Cs_2_CO_3_ and 100 nm thick Al layers were evaporated for use as the cathode. The EL spectra were measured using a PR655 spectra colorimeter. The current density–voltage and brightness–voltage curves of the devices were plotted using a Keithley 2400 source meter calibrated using a silicon photodiode. All the measurements were carried out at room temperature with no protective encapsulation. The EQE was calculated from the brightness, current density and EL spectrum assuming a Lambertian distribution.

### Synthesis of materials

#### Synthesis of 2-methoxy-9,9′-spirobi[fluorene] (MO-SP)

2-Bromo-9,9′-spirobi[fluorene] (5.0 g, 12.70 mmol) was dissolved in 50 mL DMF and then added into sodium methoxide solution (30 mL, 14.5 mmol) and cuprous iodide (2.8 g, 14.9 mmol). The reaction was heated at 120 °C in a nitrogen atmosphere for 12 h. After cooling, 100 mL water was poured into the mixture, and then dichloromethane was used three times to extract the organic solution of the crude product. The crude product was purified by silica gel column chromatography and the selected eluents were petroleum ether and dichloromethane. Finally, a white product was obtained (3.83 g, 87.2%). ^1^H-NMR (600 MHz, CDCl_3_) *δ* [ppm]: 7.84 (d, 8.4 Hz, 2H), 7.74 (d, 9.6 Hz, 2H), 7.29–7.39 (m, 3H), 7.01–7.14 (m, 3H), 6.99–7.05 (m, 1H), 6.88–6.94 (m, 1H), 6.75 (d, 8.4 Hz, 1H), 6.65 (d, 10.2 Hz, 1H), 6.21–6.27 (d, 8.4 Hz, 1H), 3.54–3.69 (s, 3H). ^13^C-NMR (150 MHz, CDCl_3_, *δ*): 160.0, 142.9, 141.9, 141.0, 133.3, 129.4, 128.7, 128.1, 126.7, 126.2, 112.8, 112.3, 63.2, 55.8. MS [*m*/*z*]: calculated for C_26_H_18_O, 346.14; found, [346.42]^+^. Elemental analysis (%): calcd for C_26_H_18_O: C, 90.14; H, 5.24. Found: C, 90.23; H, 5.31.

#### Synthesis of 9,9′-spirobi[fluoren]-2-ol (OH-SP)

2-Methoxy-9,9′-spirobi[fluorene] (2.5 g, 7.2 mmol) was dissolved in 10 mL anhydrous dichloromethane. Boron tribromide (4 g, 15.87 mmol) in 10 mL anhydrous dichloromethane was added dropwise into the solution containing 2-methoxy-9,9′-spirobi[fluorene] for 30 min at 0 °C. The reaction was completed after stirring for 4 hours at room temperature. The solution was purified by silica gel column chromatography. The solvents used were petroleum ether and dichloromethane. The white solid OH-SP was obtained (1.93 g, 81.0%). ^1^H-NMR (600 MHz, CDCl_3_) *δ*: 7.79 (d, 10.2 Hz, 2H), 7.73 (d, 8.4 Hz, 1H), 7.69 (d, 9.6 Hz, 1H), 7.29–7.39 (m, 4H), 7.07–7.13 (m, 2H), 7.00–7.06 (m, 1H), 6.66–6.84 (d, 10.2 Hz, 4H), 6.11–6.18 (m, 1H). ^13^C-NMR (150 MHz, CDCl_3_, *δ*): 156.4, 143.3, 141.9, 141.0, 133.6, 129.8, 129.2, 128.7, 128.1, 126.7, 126.2, 114.4, 113.9, 63.2. MS [*m*/*z*]: calculated for C_25_H_16_O, 332.12; found, 332.10. Elemental analysis (%): calcd for C_25_H_16_O: C, 90.33; H, 4.85. Found: C, 90.27; H, 4.92.

#### Synthesis of 4-((6-bromohexyl)oxy)-9*H*-carbazole (Br-Cz)

4-Hydroxyl-9*H*-carbazole (3.0 g, 16.4 mmol), 1,6-dibromohexane (16.0 g, 65.6 mmol) and cesium carbonate (20 g, 61.3 mmol) were taken in a 50 mL bottle, then 20 mL DMF was poured into it, and then the mixture was stirred under nitrogen for 90 min. After that, the mixture was poured into 100 mL water to precipitate the product. After separation and purification using a chromatography column with petroleum ether and dichloromethane, a white solid was obtained (3.1 g, yield 55.0%). ^1^H-NMR (600 MHz, CDCl_3_) *δ*: 8.26–8.34 (d, 1H), 7.94–8.04 (s, 1H), 7.20–7.44 (m, 4H), 6.97–7.04 (d, 1H), 6.60–6.67 (d, 1H), 4.17–4.27 (m, 2H), 3.36–3.48 (m, 2H), 1.52–2.08 (m, 8H). ^13^C-NMR (150 MHz, CDCl_3_, *δ*): 149.0, 141.3, 130.2, 128.3, 124.1, 121.7, 121.4, 121.3, 119.8, 111.1, 107.6, 102.7, 69.0, 33.7, 32.6, 29.6, 25.4, 24.9. MS (MALDI-TOF) [*m*/*z*]: calculated for C_18_H_20_BrNO, 345.07; found, 345.11. Elemental analysis. Calcd for C_18_H_20_BrNO: C, 62.44; H, 5.82; Br, 23.08; N, 4.05. Found: C, 62.28; H, 5.64; Br, 23.21; N, 4.01.

#### Synthesis of 4-((6-(9,9'-spirobi[fluoren]-2-yloxy)hexyl)oxy)-9H-carbazole (Cz-SP)

A mixture of OH-SP (1.8 g, 5.4 mmol) and Br-Cz (2.1 g, 6.1 mmol) was taken in a 50 mL bottle, and then cesium carbonate (Cs_2_CO_3_, 10 g, 30.7 mmol) was added into it; 15 mL DMF was added into the mixture and stirred for 1.5 h at room temperature under nitrogen, and then 100 mL water was poured into the mixture and extracted three times with CH_2_Cl_2_. Then the combined organic layer was dried over anhydrous NaSO_4_, and the solvent was removed under vacuum. Finally, the crude product was purified by column chromatography on silica gel, and a white product was obtained (3.06 g, 95%). ^1^H-NMR (600 MHz, CDCl_3_) *δ*: 11.21 (s, 1H), 8.26 (d, 7.2 Hz, 1H), 7.99 (s, 1H), 7.80–7.84 (m, 2H), 7.04–7.76 (m, 2H), 7.27–7.38 (m, 6H), 7.17–7.20 (m, 1H), 7.07–7.14 (m, 2H), 6.98–7.05 (m, 2H), 6.89 (d, 7.8 Hz, 1H), 6.75 (d, 7.2 Hz, 1H), 6.62 (d, 8.4 Hz, 2H), 6.25 (d, 7.2 Hz, 1H), 4.14–4.21 (m, 2H), 3.75–3.80 (m, 2H), 1.92–1.98 (m, 2H), 1.45–1.74 (m, 6H). ^13^C-NMR (150 MHz, CDCl_3_, *δ*): 156.8, 149.0, 142.5, 141.9, 141.3, 141.0, 132.6, 130.2, 129.0, 128.7, 128.1, 126.7, 126.2, 119.8, 112.9, 112.4, 111.1, 107.6, 102.7, 69.0, 68.7, 29.6, 25.9. MS (MALDI-TOF) [*m*/*z*]: calculated for C_43_H_35_NO_2_, 597.26; found, 597.47. Elemental analysis. Calcd for C_43_H_35_NO_2_: C, 86.4; H, 5.90; N, 2.34. Found: C, 86.43; H, 5.89; N, 2.32.

#### Synthesis of 2,3,4,5,6-pentakis(4-((6-(9,9'-spirobi[fluoren]-2-yloxy)hexyl)oxy)-9H-carbazol-9-yl)benzonitrile (5CzBN-PSP)

Cz-SP (1.0 g, 1.67 mmol) was taken in anhydrous THF (10 mL), then sodium hydride was added (0.14 g, 6.01 mmol) into the mixture, and stirred in a nitrogen atmosphere for 0.5 h. Then 2,3,4,5,6-pentafluoronitrile (0.054 g, 0.28 mmol) was slowly added into it. Under a nitrogen atmosphere the mixture was stirred at room temperature for 72 h. After that, water was added slowly to quench the excess NaH, and then, 200 mL of water was added into the solution mixture and extracted three times with CH_2_Cl_2_. The organic liquid was dried with anhydrous sodium sulfate and the solvent was removed under vacuum. The final product was separated by silica gel column chromatography. Finally, a bright green solid powder was obtained (0.51 g, yield 50%). ^1^H-NMR (600 MHz, CDCl_3_) *δ*: 7.90 (s, 2H), 7.57–7.81 (m, 20H), 7.41–7.51 (m, 3H), 7.26–7.34 (m, 15H), 6.98–7.18 (m, 23H), 6.89–6.94 (m, 4H), 6.65–6.85 (m, 27H), 6.37–6.61 (m, 8H), 6.13–6.23 (m, 5H), 6.05 (d, 12.0 Hz, 3H), 3.54–4.00 (m, 20H), 1.38–1.81 (m, 40H). ^13^C-NMR (150 MHz, CDCl_3_, *δ*): 159.4, 156.2, 150.5, 150.0, 148.2, 145.4, 141.6, 134.4, 127.8, 127.7, 126.5, 124.1, 120.6, 119.9, 119.1, 116.6, 114.1, 110.0, 102.5, 67.8, 66.0, 34.7, 29.1, 29.0, 26.8, 26.0, 25.7. MS (MALDI-TOF) [*m*/*z*]: calculated for C_222_H_170_N_6_O_10_, 3081.30; found, 3081.84. Elemental analysis. Calcd for C_222_H_170_N_6_O_10_: C, 86.52; H, 5.56; N, 2.73. Found: C, 86.53; H, 5.55; N, 2.72.

#### Synthesis of 3,5-bis(4-((6-(9,9′-spirobi[fluoren]-2-yloxy)hexyl)oxy)-9*H*-carbazol-9-yl)-2,4,6-tri(9*H*-carbazol-9-yl)benzonitrile (5CzBN-DSP)

The synthesis procedure for this compound is the same as that for 5CzBN-PSP (0.51 g, 57%). ^1^H-NMR (600 MHz, CDCl_3_) *δ*: 7.69–8.02 (m, 19H), 7.25–7.44 (m, 12H), 7.02–7.15 (m, 14H), 6.88–6.93 (m, 2H), 6.53–6.60 (m, 15H), 6.23 (d, 10.2 Hz, 3H), 6.05 (s, 2H), 5.75 (s, 1H), 3.64–3.82 (m, 8H), 1.35–1.63 (m, 16H). ^13^C-NMR (150 MHz, CDCl_3_, *δ*):159.3, 154.6, 150.6, 149.0, 148.4, 141.5, 140.4, 139.3, 137.9, 136.7, 134.5, 127.8, 126.6, 125.3, 124.1, 123.8, 120.7, 120.0, 119.0, 116.8, 114.0, 110.0, 109.7, 103.4, 67.8, 67.4, 66.0, 28.8, 26.9, 25.4. MS (MALDI-TOF) [*m*/*z*]: calculated for C_129_H_92_N_6_O_4_, 1788.72; found, 1788.73. Elemental analysis. Calcd for C_129_H_92_N_6_O_4_: C, 86.55; H, 5.18; N, 4.69. Found: C, 86.58; H, 5.15; N, 4.68.

#### Synthesis of 3-(4-((6-(9,9′-spirobi[fluoren]-2-yloxy)hexyl)oxy)-9*H*-carbazol-9-yl)-2,4,5,6-tetra(9*H*-carbazol-9-yl)benzonitrile (5CzBN-SSP)

The synthesis procedure for this compound is the same as that for 5CzBN-PSP (0.47 g, 52%). ^1^H-NMR (600 MHz, CDCl_3_) *δ*: 7.64–8.02 (m, 17H), 7.27–7.45 (m, 9H), 6.98–7.18 (m, 11H), 6.88–6.94 (m, 1H), 6.47–6.76 (m, 14H), 6.21–6.26 (m, 1H), 5.99–6.06 (m, 1H), 3.67–3.85 (m, 4H), 1.35–1.64 (m, 8H). ^13^C-NMR (150 MHz, CDCl_3_, *δ*): 159.3, 154.6, 150.5, 149.0, 148.4, 141.8, 140.5, 139.2, 139.0, 137.7, 137.0, 136.4, 128.0, 127.8, 126.5, 125.3, 124.1, 124.0, 123.9, 122.2, 121.1, 120.9, 120.5, 120.0, 119.4, 116.9, 114.0, 113.0, 112.7, 110.3, 109.6, 103.4, 102.8, 67.8, 67.4, 65.8, 29.1, 28.9, 26.9, 25.7. MS (MALDI-TOF) [*m*/*z*]: calculated for C_98_H_66_N_6_O_2_, 1358.52; found, 1358.33. Elemental analysis. Calcd for C_98_H_66_N_6_O_2_: C, 86.57; H, 4.89; N, 6.18. Found: C, 86.55; H, 4.91; N, 6.16.

## Conflicts of interest

There are no conflicts to declare.

## Supplementary Material

Supplementary information
