# Supplementary material for: Endowing TADF luminophors with AIE properties through adjusting flexible dendrons for highly efficient solution-processed nondoped OLEDs
Source: Chem Sci. 2020 Jun 16;11(27):7194–203. doi: 10.1039/d0sc02194f (PMC7499814; doi:10.1039/d0sc02194f)
Supplement: Supplementary file 1 [file SC-011-D0SC02194F-s001.pdf]

Electronic Supplementary Material (ESI) for Chemical Science.
This journal is © The Royal Society of Chemistry 2020

Supporting Information

Endowing TADF Luminophors with AIE Properties Though Adjusting Flexible Dendrons for Highly Efficient Solution-Processed Non-doped OLEDs.

Dan Liu, [† a] Jing Yi Wei, [† a] Wen Wen Tian, [a] Wei Jiang* [a], Yue Ming Sun,[a] Zheng Zhao[b] and Ben Zhong Tang*[b]

[a]Jiangsu Province Hi-Tech Key Laboratory for Bio-Medical Research, Jiangsu Engineering Laboratory of Smart Carbon-Rich Materials and Device, School of Chemistry and Chemical Engineering, Southeast University, Nanjing, Jiangsu, 211189, P. R. China.

[b]Department of Chemistry, Hong Kong Branch of Chinese National Engineering Research, Center for Tissue Restoration and Reconstruction, The Hong Kong University of Science and Technology, Clear Water Bay, Kowloon, Hong Kong 999077, China.

[†] Dan Liu and Jing Yi Wei are contributed equally to this work.

*Corresponding author: Wei Jiang, jiangw@seu.edu.cn

Ben Zhong Tang, tangbenz@ust.hk

KEYWORDS: Thermally activated delayed fluorescence (TADF), Aggregation-induced emission (AIE), Core-dendron system, solution-processed, Organic light-emitting diodes (OLED).

Figure Captions

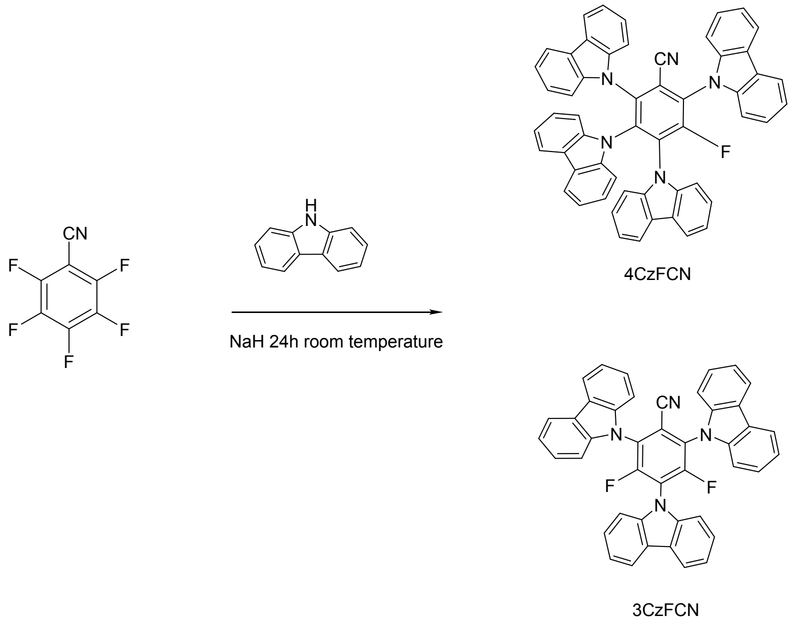

Scheme S1 Synthetic routes of 4CzFCN and 5CzFCN

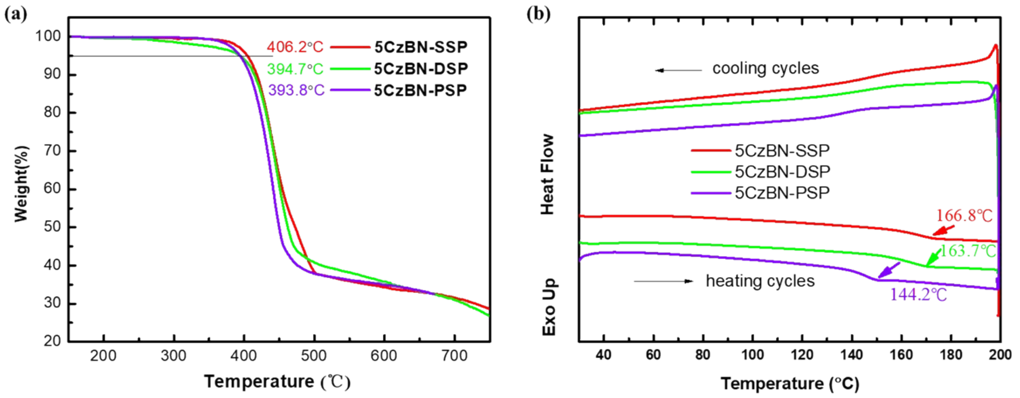

Figure S1 TGA and DSC traces of 5CzBN-SSP, 5CzBN-DSP and 5CzBN-PSP recorded at a heating rate of 10℃/min.

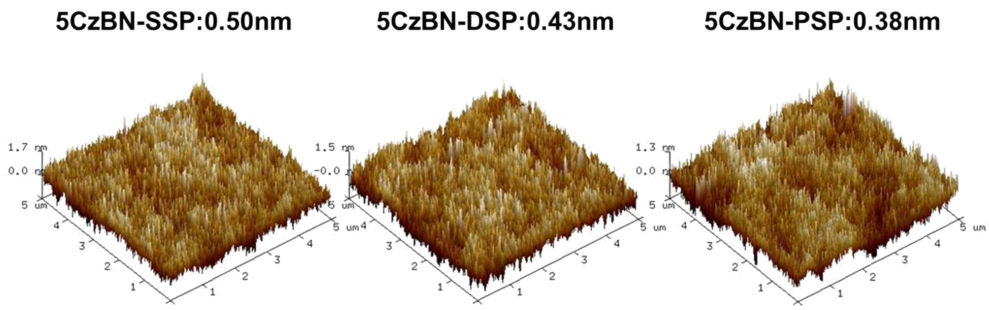

Figure S2 AFM topographic images of 5CzBN-SSP, 5CzBN-DSP and 5CzBN-PSP.

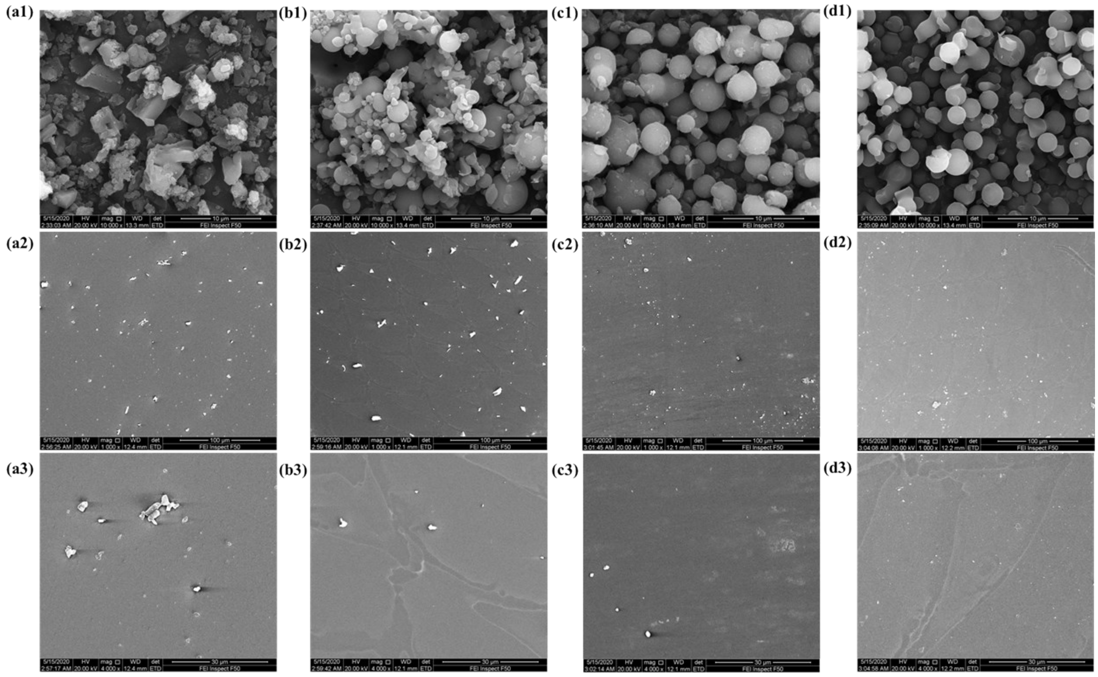

Figure S3 The SEM images of (a)5CzBN, (b)5CzBN-SSP, (c) 5CzBN-DSP and (d) 5CzBN-PSP, where a1-d1 were in the solid states, a2-d2 and a3-d3 were in solution-processed film states at various magnifications of 1000 and 4000, respectively.

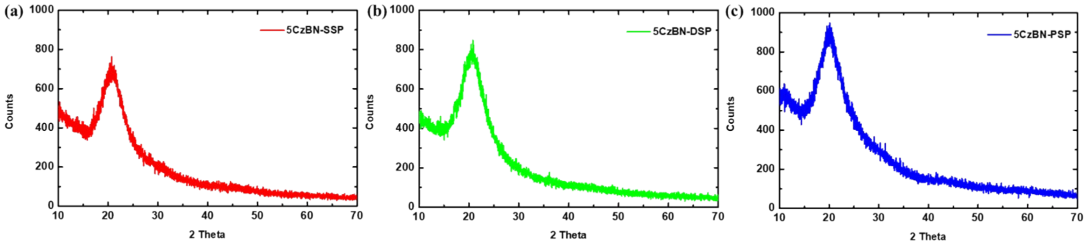

Figure S4 The XRD plots of 5CzBN-SSP, 5CzBN-DSP and 5CzBN-PSP.

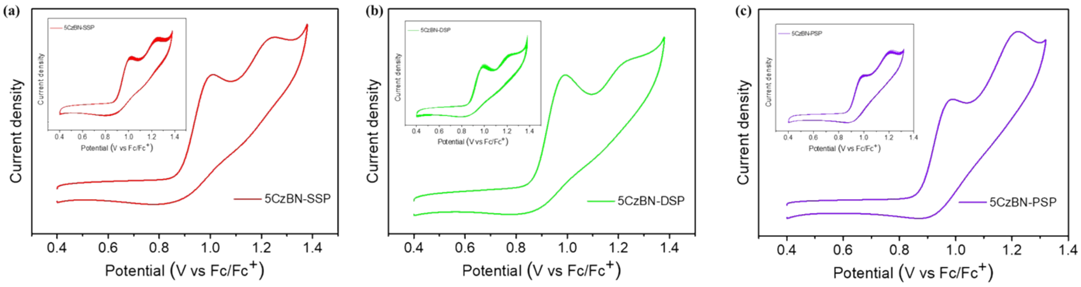

Figure S5 Cyclic voltammogram of 5CzBN-SSP (a), 5CzBN-DSP (b) and 5CzBN-PSP(c) after one circle and 100 circles (inset).

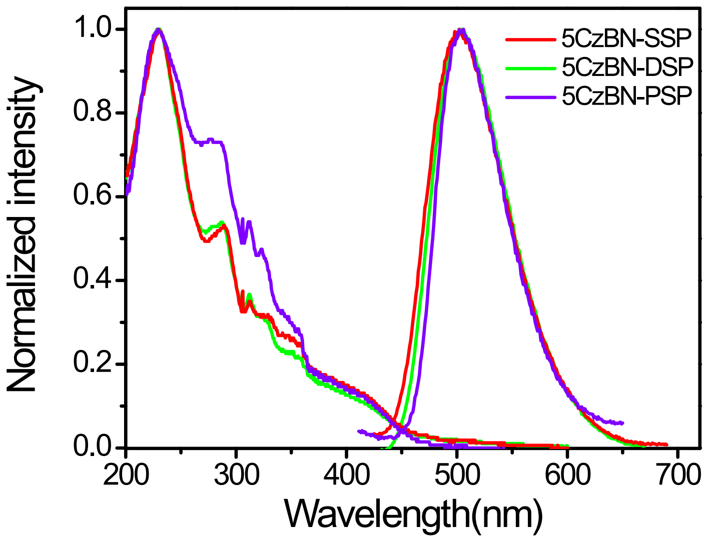

Figure S6 UV-vis absorption and fluorescence spectra of 5CzBN-SSP, 5CzBN-DSP and 5CzBN-PSP in spin-coated films.

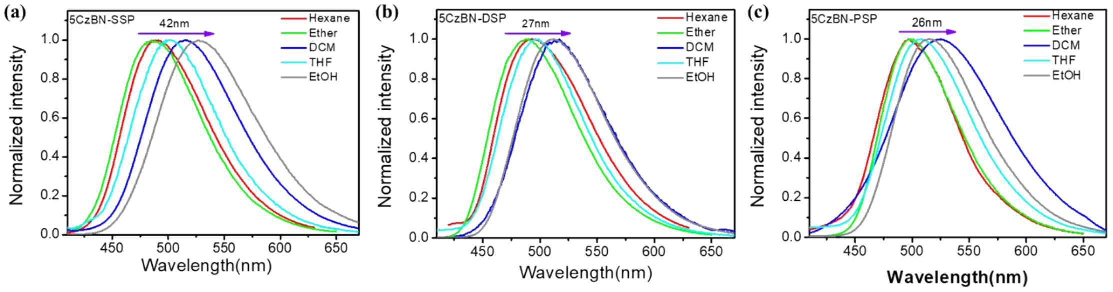

Figure S7 The PL spectra of (a) 5CzBN-SP, (b) 5CzBN-DSP, (c) 5CzBN-QSP in hexane, ether, CH_2Cl_2, THF and EtOH.

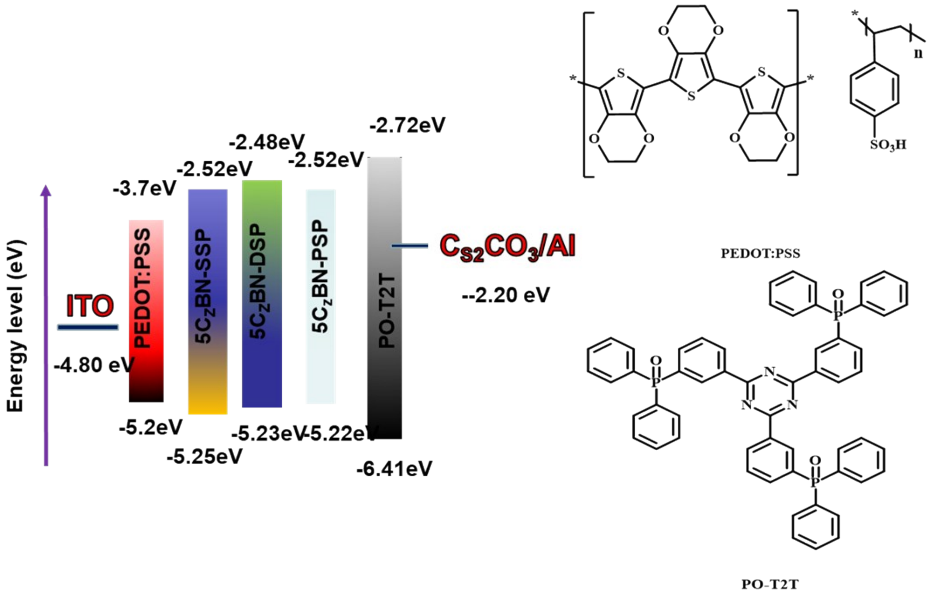

Figure S8 schematic energy-level diagrams and the fabrication process of the devices

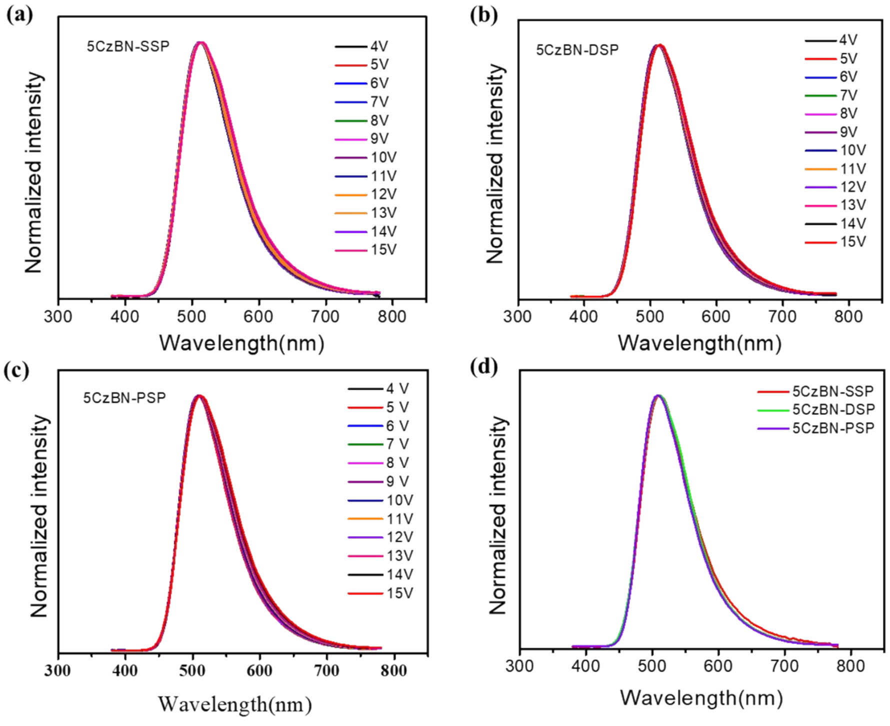

Figure S9 (a) EL spectra of 5CzBN-SSP in various voltages, (b) EL spectra of 5CzBN-DSP in various voltages, (c) EL spectra of 5CzBN-PSP in various voltages, (d) EL spectra of three materials at 10 V.

Table S1 Comparison of EL performance of solution-processed nondoped OLEDs based on AIE materials.

	Solution-processed EML	EL (nm)	CE_{max} [cd A^{-1}]	PE_{max} [lm W^{-1}]	EQE_{max}[%]	CIE(x,y)	Reference
Fully solution process	5CzBN-PSP	508	58.7	46.2	20.1	(0.28,0.54)	This work
	G2B	500	14.0	11.5	5.7	(0.26,0.48)	[47]
	G3B	516	7.7	5.7	2.9	(0.31,0.50)	[47]
ETL evaporation	TPEF	478	4.55	-	2.17	(0.21,0.32)	[48]
	TPE-2PN2PB	543	8.3	7.5	2.6	(0.37,0.54)	[49]
	TPE-DFCz	500	1.16	-	0.46	(0.24,0.43)	[50]
	p-DTPACO	517	10.8	8.2	3.7	(0.33,0.51)	[51]
	Cz-AQ	600/680	-	-	0.75/1.15	-	[52]
	G2B	500	11.3	7.1	4.8	(0.25,0.44)	[47]
	SFC	536	3.67	-	1.37	(0.36,0.52)	[53]
	CzTAZP	515	23.1	-	9.4	(0.27,0.58)	[54]
	m-ACSO2	486	37.9	23.8	17.2	(0.21,0.34)	[55]
	BPCP-2CPC	524	40.8	28.5	13.4	(0.33,0.54)	[32]
	Ac3TRZ3	520	11.4	-	3.5	(0.30,0.54)	[56]
	CzTAZPO	537	29.1	-	12.8	(0.37,0.56)	[57]
	3CzTPE	479	4.35	-	2.81	(0.18,0.29)	[58]

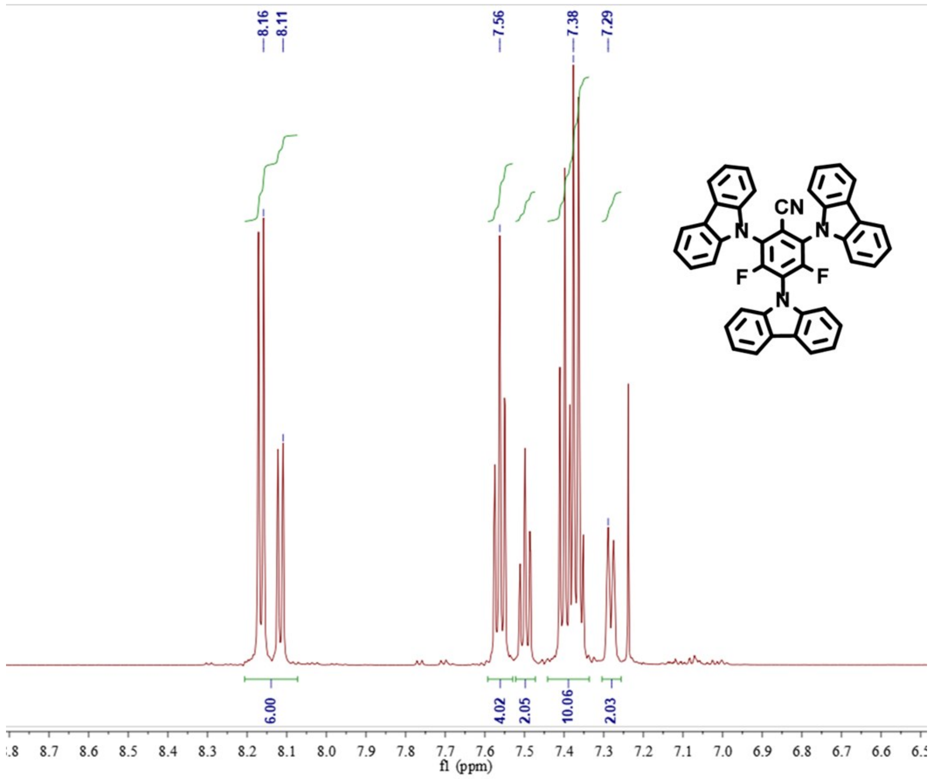

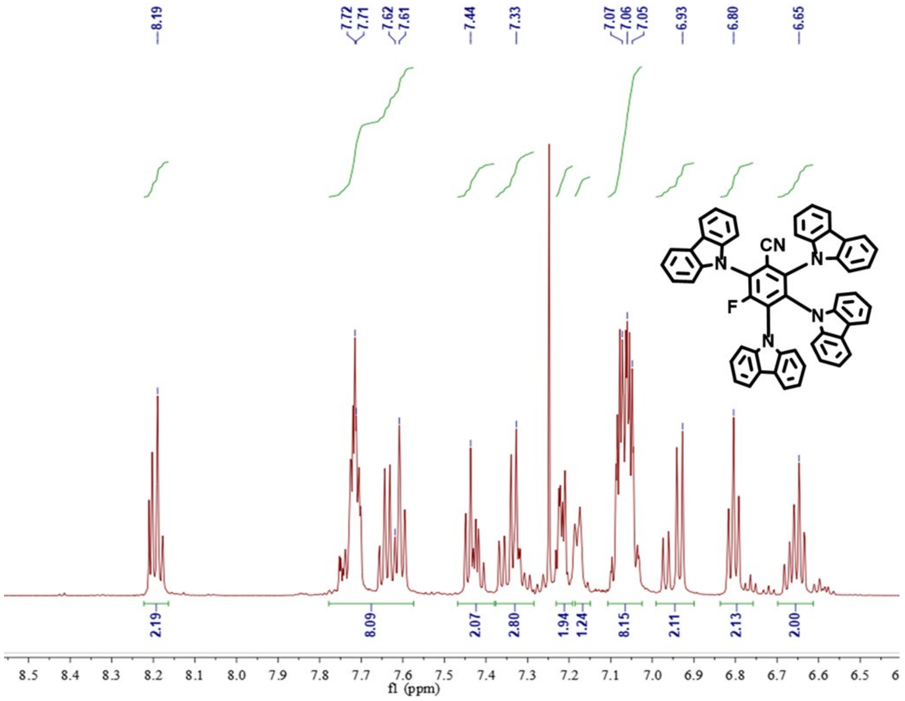

8.19
7.72
7.71
7.62
7.61
7.44
7.33
7.07
7.06
7.05
6.93
6.80
6.65
CN
F
N
2.19
8.09
2.07
2.80
1.94
1.24
8.15
2.11
2.13
2.00
8.5 8.4 8.3 8.2 8.1 8.0 7.9 7.8 7.7 7.6 7.5 7.4 7.3 7.2 7.1 7.0 6.9 6.8 6.7 6.6 6.5 6
f1 (ppm)

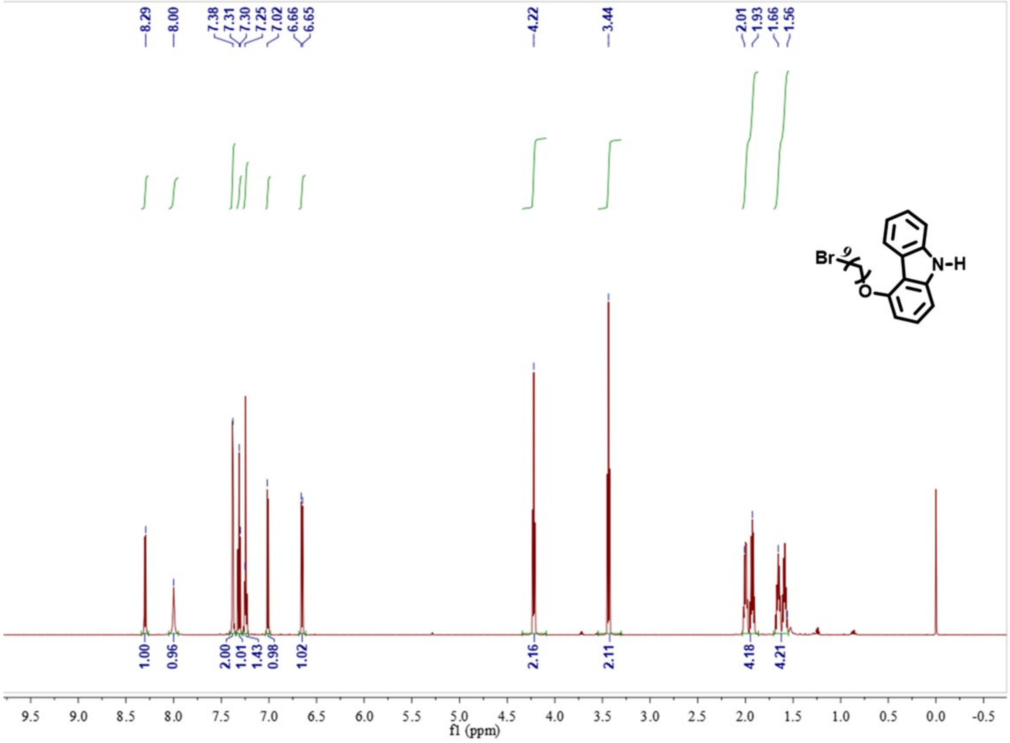

8.29
8.00
7.38
7.31
7.30
7.25
7.02
6.66
6.65
4.22
3.44
2.01
1.93
1.66
1.56
Br
9
O
N-H
1.00
0.96
2.00
1.01
1.43
0.98
1.02
2.16
2.11
4.18
4.21
9.5 9.0 8.5 8.0 7.5 7.0 6.5 6.0 5.5 5.0 4.5 4.0 3.5 3.0 2.5 2.0 1.5 1.0 0.5 0.0 -0.5
f1 (ppm)

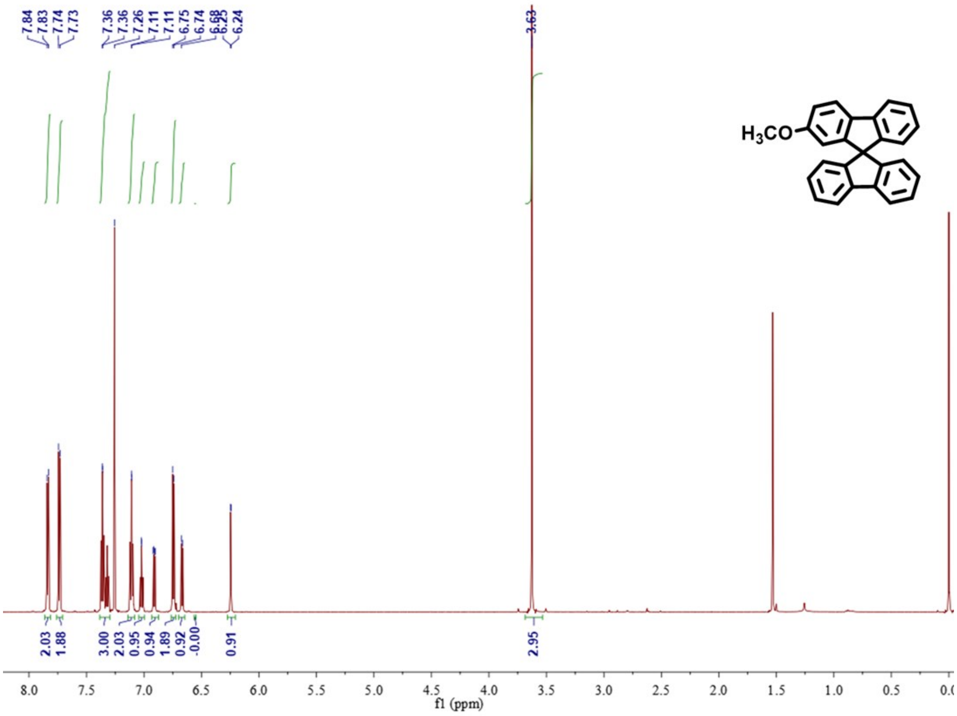
7.84
7.83
7.74
7.73
7.36
7.36
7.26
7.11
7.11
6.75
6.74
6.68
6.25
6.24
3.63
H3CO
2.03
1.88
3.00
2.03
0.95
0.94
1.89
0.92
-0.00
0.91
2.95
8.0 7.5 7.0 6.5 6.0 5.5 5.0 4.5 4.0 3.5 3.0 2.5 2.0 1.5 1.0 0.5
f1 (ppm)

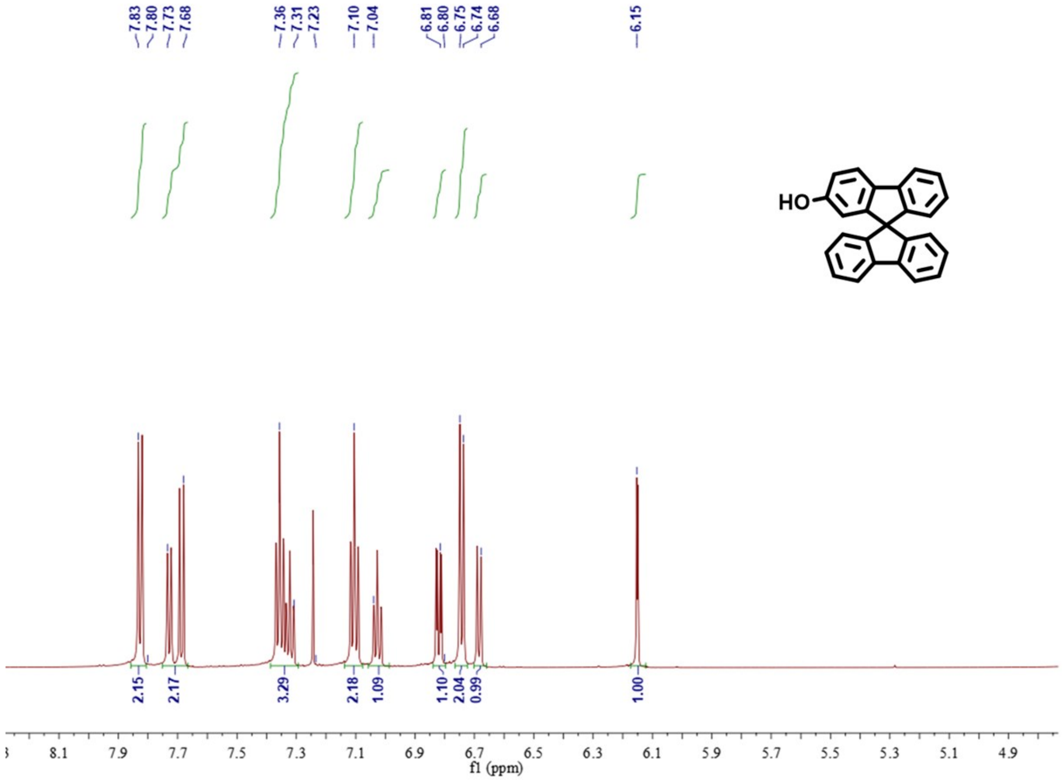
7.83
7.80
7.73
7.68
7.36
7.31
7.23
7.10
7.04
6.81
6.80
6.75
6.74
6.68
6.15
HO
2.15
2.17
3.29
2.18
1.09
1.10
2.04
0.99
1.00
8.1 7.9 7.7 7.5 7.3 7.1 6.9 6.7 6.5 6.3 6.1 5.9 5.7 5.5 5.3 5.1 4.9
f1 (ppm)

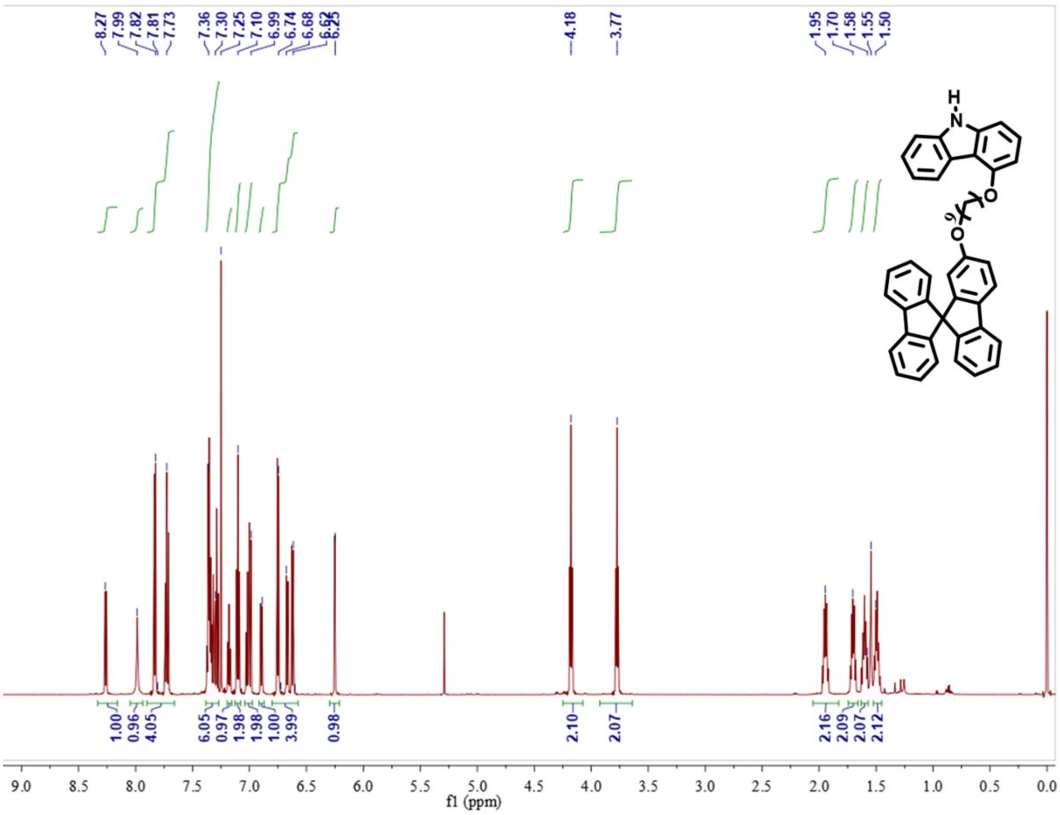
8.27 7.99 7.82 7.81 7.73 7.36 7.30 7.25 7.10 6.99 6.74 6.68 6.62 6.25
4.18 3.77
1.95 1.70 1.58 1.55 1.50
1.00 0.96 4.05 6.05 0.97 1.98 1.98 1.00 3.99 0.98 2.10 2.07 2.16 2.09 2.07 2.12
9.0 8.5 8.0 7.5 7.0 6.5 6.0 5.5 5.0 4.5 4.0 3.5 3.0 2.5 2.0 1.5 1.0 0.5 0.0
f1 (ppm)

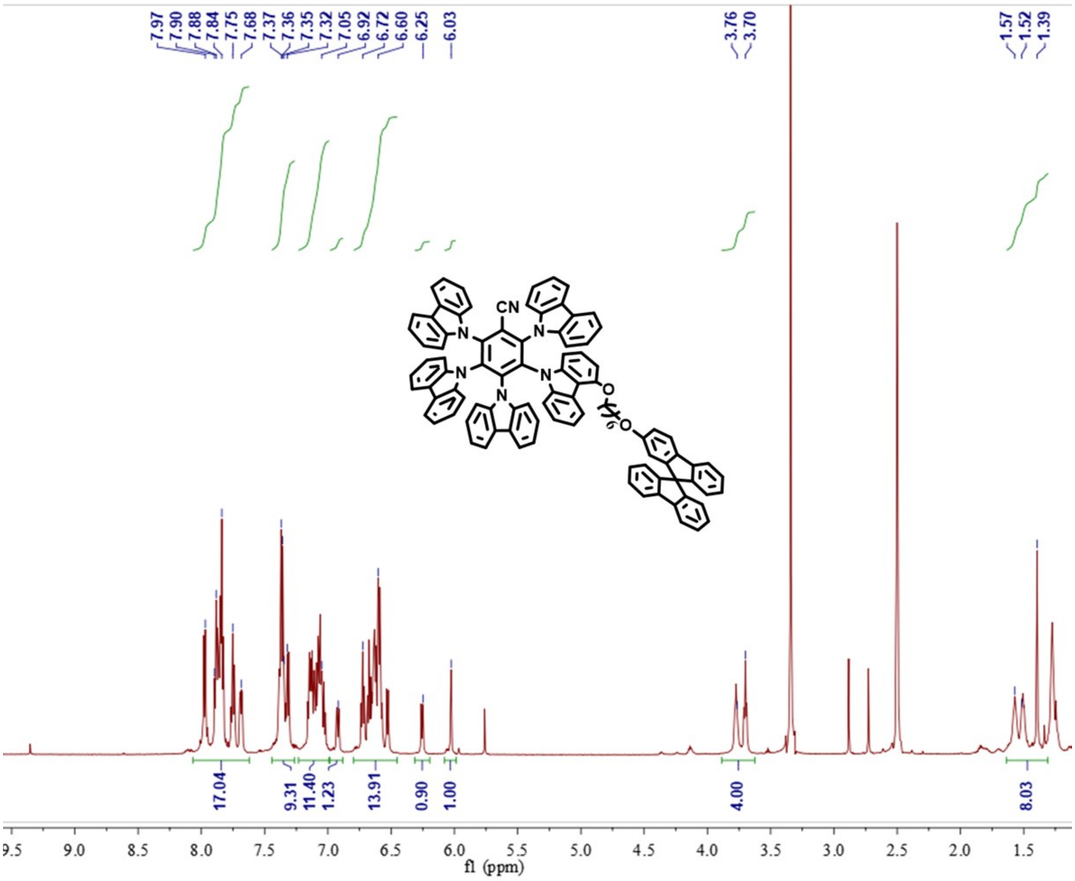
7.97 7.90 7.88 7.84 7.75 7.68 7.37 7.36 7.35 7.32 7.05 6.92 6.72 6.60 6.25 6.03
3.76 3.70
1.57 1.52 1.39
CN
17.04 9.31 11.40 1.23 13.91 0.90 1.00 4.00 8.03
9.5 9.0 8.5 8.0 7.5 7.0 6.5 6.0 5.5 5.0 4.5 4.0 3.5 3.0 2.5 2.0 1.5
f1 (ppm)

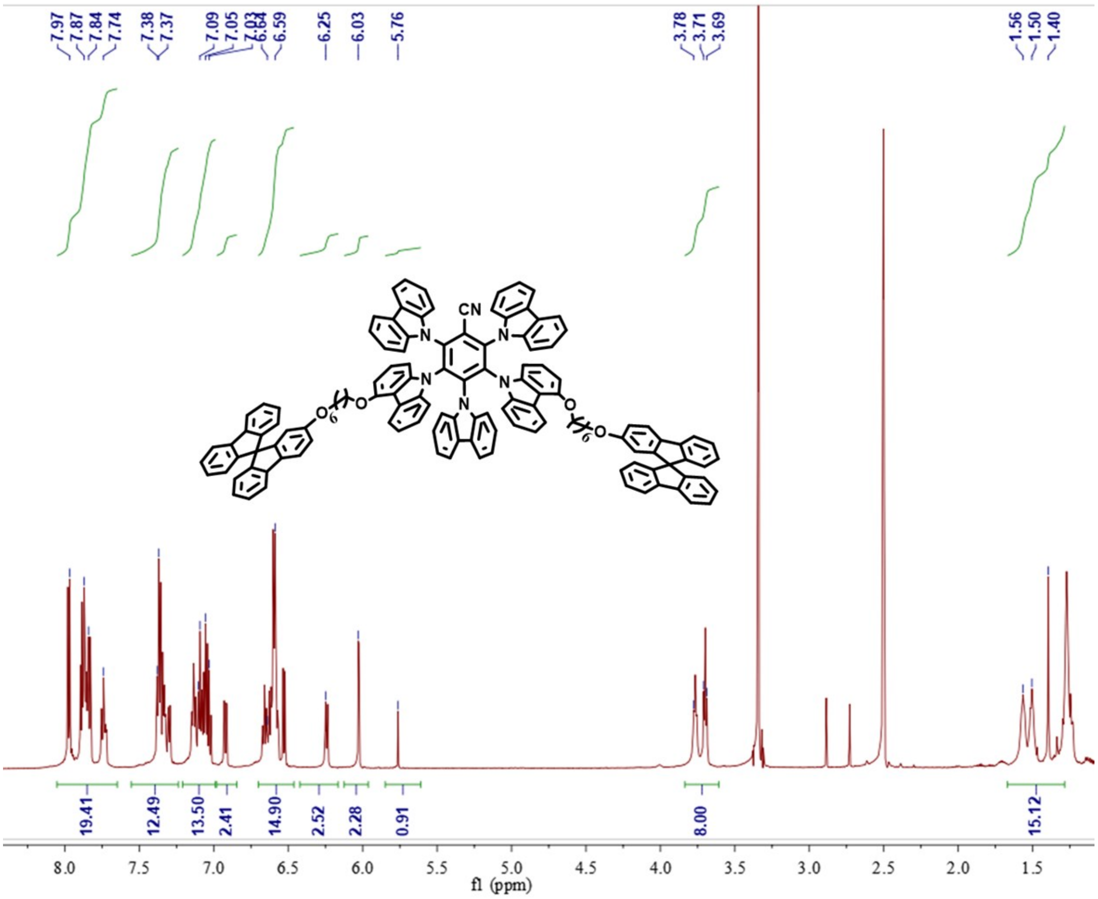
f1 (ppm)

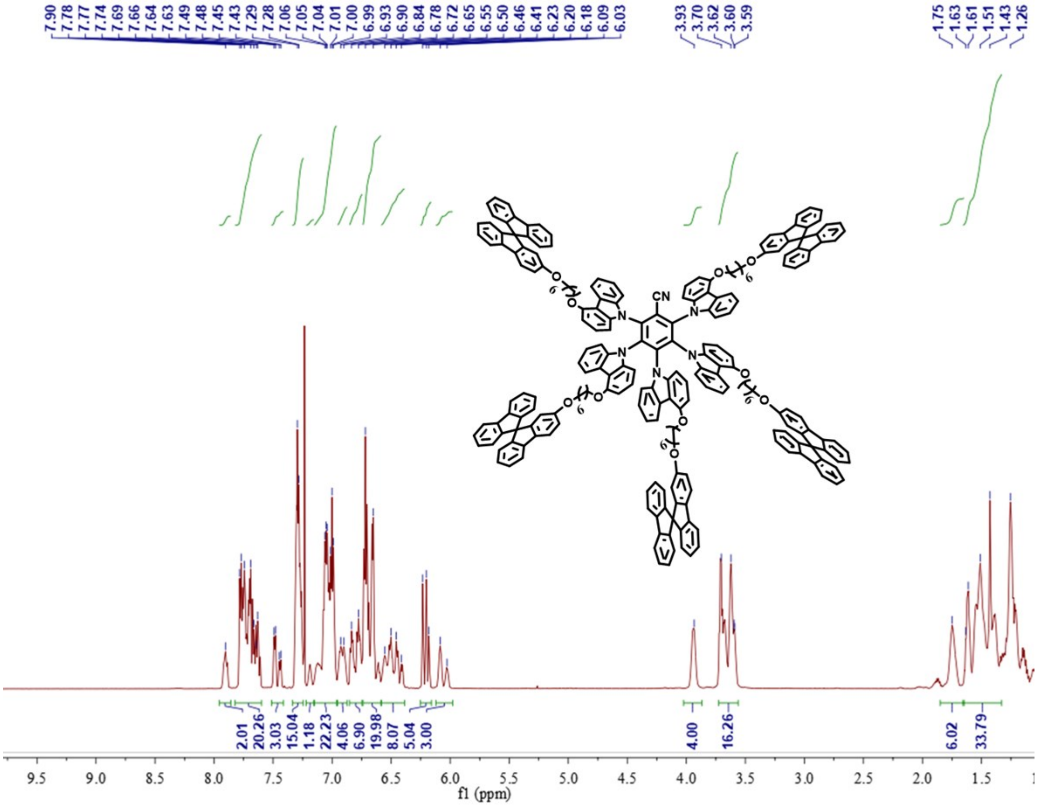
f1 (ppm)

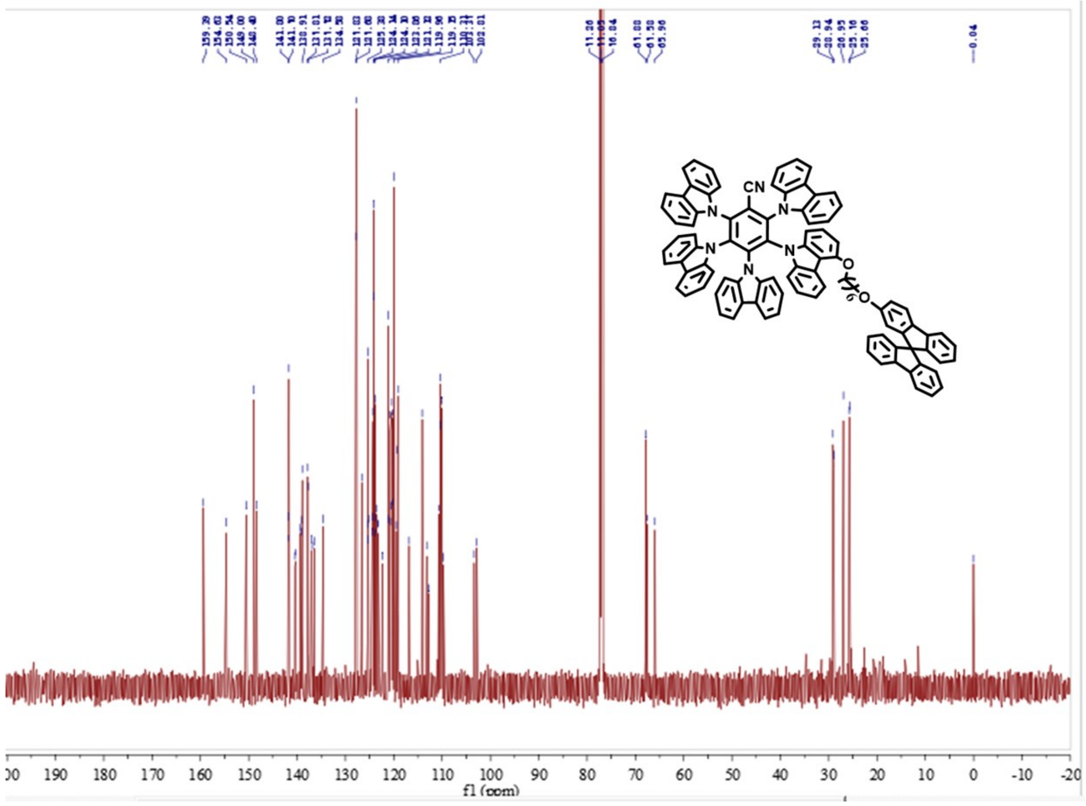

CN
N
O
6
O
00 190 180 170 160 150 140 130 120 110 100 90 80 70 60 50 40 30 20 10 0 -10 -20
f1 (ppm)

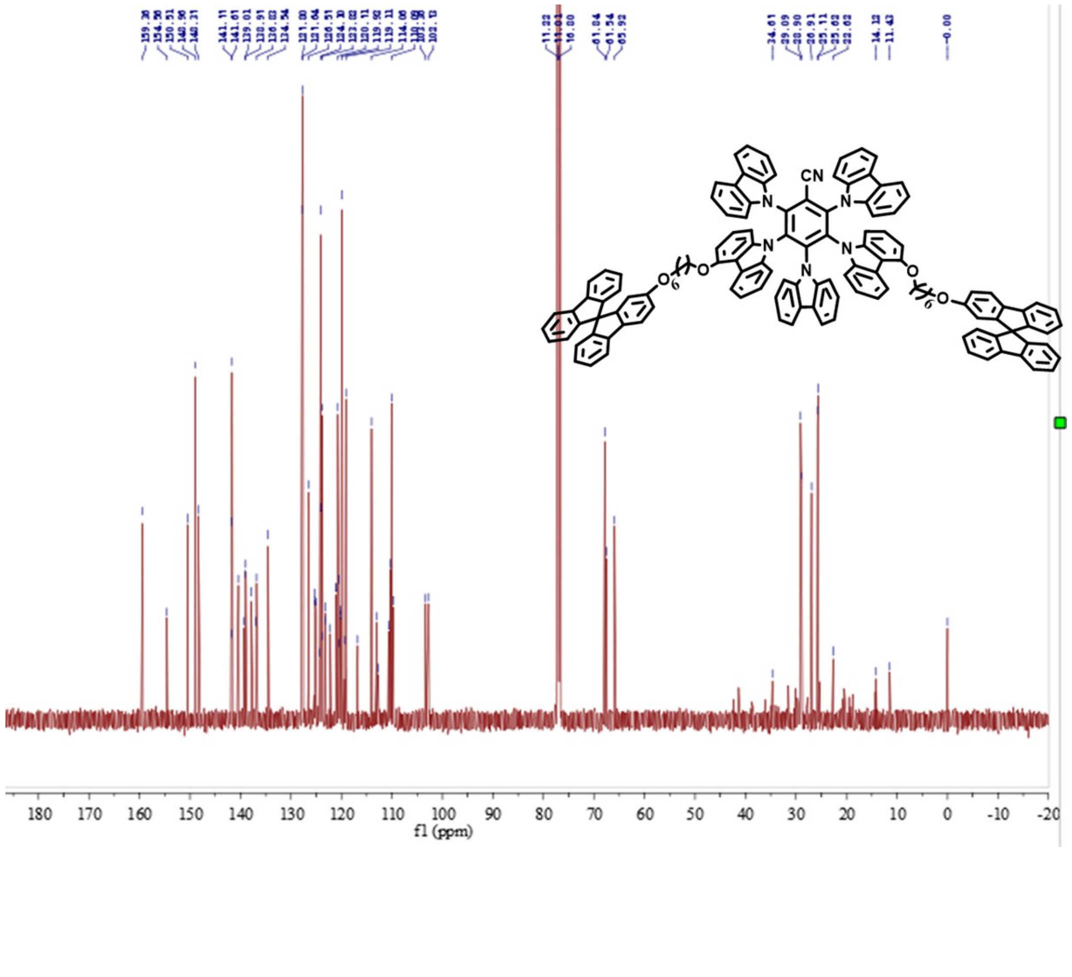

CN
N
O
6
O
180 170 160 150 140 130 120 110 100 90 80 70 60 50 40 30 20 10 0 -10 -20
f1 (ppm)

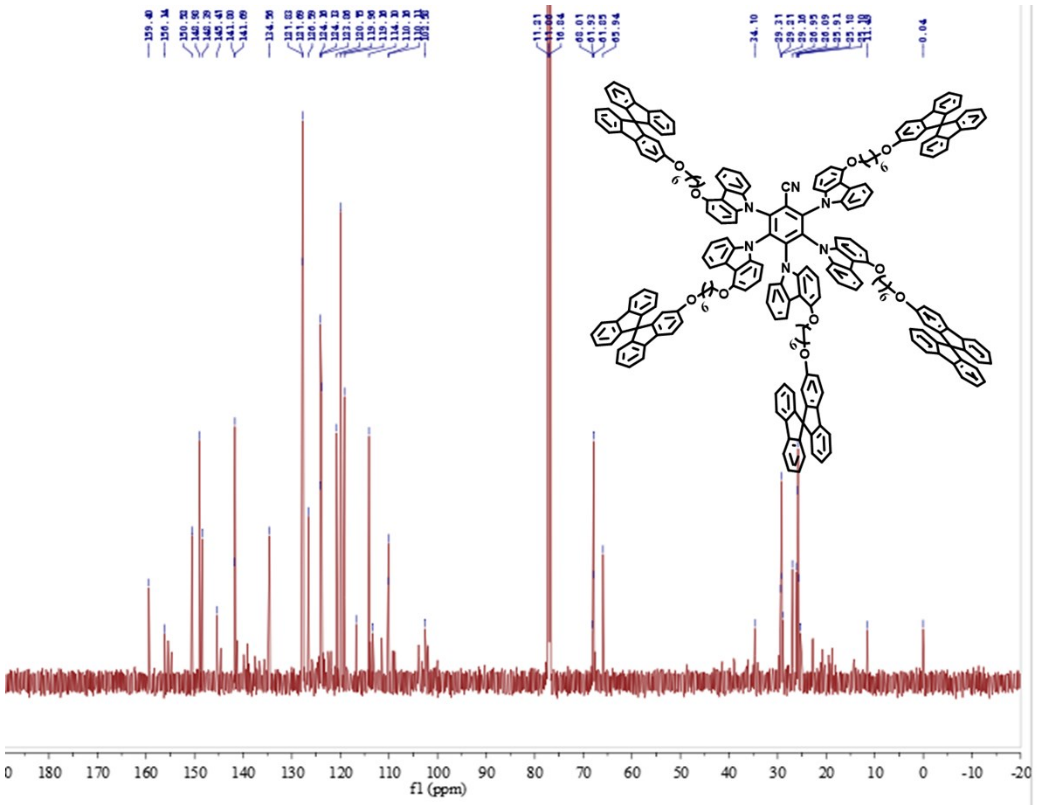

f1 (ppm)
CN
N
O
6
